# Phytoliths in Inflorescence Bracts: Preliminary Results of an Investigation on Common Panicoideae Plants in China

**DOI:** 10.3389/fpls.2019.01736

**Published:** 2020-02-20

**Authors:** Yong Ge, Houyuan Lu, Jianping Zhang, Can Wang, Xing Gao

**Affiliations:** ^1^Key Laboratory of Vertebrate Evolution and Human Origins, Institute of Vertebrate Paleontology and Paleoanthropology, Chinese Academy of Sciences, Beijing, China; ^2^Center for Excellence in Life and Paleoenvironment, Chinese Academy of Sciences, Beijing, China; ^3^Key Laboratory of Cenozoic Geology and Environment, Institute of Geology and Geophysics, Chinese Academy of Sciences, Beijing, China; ^4^Center for Excellence in Tibetan Plateau Earth Science, Chinese Academy of Sciences, Beijing, China; ^5^University of Chinese Academy of Sciences, Beijing, China; ^6^Department of Archaeology, School of History and Culture, Shandong University, Jinan, China

**Keywords:** phytolith morphology, Poaceae taxonomy, inflorescence phytolith, seed protection strategy, archaeobotanical implication

## Abstract

Phytoliths in the inflorescence of Poaceae plants can be of high taxonomic value in some archaeological contexts and provide insight into plant taxonomy and crop domestication processes. In this study, phytoliths in every inflorescence bract of 38 common Panicoideae weeds and minor crops in China were studied. Based on dissection of the inflorescence into different bracts using a treatment that retained the phytoliths anatomical position, observations of inflorescence phytoliths types and distribution were described in detail. We found that Interdigitating, Blocky amoeboid, Rectangular dentate, and Elongate dendritic with multi tent-like arch tops were of higher taxonomic value than the other types in our studied species. Both morphological and morphometric traits of the Interdigitating were summarized and compared with previous studies; the findings suggested that genus level discrimination of some Paniceae species could be reliable, and tribe/species level discrimination might be feasible. The phytoliths in the involucre of domesticated and wild type *Coix lacryma-jobi* provided insight into the domestication process of this plant. Our data also indicated that phytolith production in the inflorescence bracts might be under the genetic and molecular control of inflorescence development. Thus, the findings of this study could assist future studies in plant taxonomy and archaeobotany.

## Introduction

Phytoliths are plant-produced micro silica bodies which can, in some cases, have a diagnostic morphology that can be distinguished among taxa; in particular, phytoliths in the Poaceae family can have taxonomic value in archaeological contexts and natural sediments (Twiss et al., 1969; Wang and Lu, 1993; Piperno, 2006). Based on phytolith taxonomy and morphology, phytolith analysis has been proven to be a reliable tool in understanding the taxonomy and evolution of plants (Prychid et al., 2003; Rudall et al., 2014; Dinda and Mondal, 2018), paleoecology (Blinnikov et al., 2002; Stromberg, 2005; Gu et al., 2008; Stromberg et al., 2013; Dunn et al., 2015), and paleoclimate (Prebble and Shulmeister, 2002; Lu et al., 2007; Zuo et al., 2016; Liu H. et al., 2018); in recent years, it has been extensively employed in investigating the origin, development, and spread of agriculture (Lu et al., 2009a; Piperno et al., 2009; Madella et al., 2014; Ball et al., 2016a; Hilbert et al., 2017; Deng et al., 2018; He et al., 2018). However, compared to studies on the leaf phytoliths of Poaceae plants, inflorescence phytoliths have not been extensively studied and have generally focused on crop species and their relatives (Ball et al., 2016a). Thus, a broad and systematic investigation of inflorescence phytoliths would provide an important advancement and enable multidisciplinary application of phytolith analysis.

The study of inflorescence phytoliths has a long history; as early as 1908, Schellenberg studied inflorescence phytoliths in archaeological contexts (Schellenberg, 1908), and in 1966, Parry and Smithson studied the inflorescence phytoliths of grasses and cereals in Britain using an acid treatment to extract the phytoliths and observing them under a light microscope (Parry and Smithson, 1966). Thereafter, scanning electron microscopy was introduced to study inflorescence phytoliths (Terrell and Wergin, 1981; Sangster et al., 1983; Rosen and Weiner, 1994). With the introduction of phytolith analysis among archaeologists, Poaceae inflorescence phytoliths were valued due to their relationship with human food gathering activities, and the inflorescence phytoliths of both major (Hodson and Sangster, 1988; Piperno and Pearsall, 1993; Tubb et al., 1993; Pearsall et al., 1995; Zhao et al., 1998; Ball et al., 1999; Rosen, 1999; Ball et al., 2001; Pearsall et al., 2003; Rosen, 2004; Hodson et al., 2008; Madella et al., 2014; Ball et al., 2017) and minor (Lu et al., 2009b; Radomski and Neumann, 2011; Zhang et al., 2011; Madella et al., 2013; Kealhofer et al., 2015; Novello and Barboni, 2015; Weisskopf and Lee, 2016; Ge et al., 2018; Zhang et al., 2018; Duncan et al., 2019) crops were extensively studied to investigate crop domestication. Nevertheless, many other wild relatives and minor crops have not been studied, which not only results in identification uncertainty, but also has stalled further application of phytolith analysis in the investigation of early plant resource exploitation.

Panicoideae plants such as foxtail millet (*Setaria italica*), common millet (*Panicum miliaceum*), and barnyard millet (*Echinocloa* sp.) are widely recognized as minor crops that could have been important plant resources in ancient times (Bellwood, 2004; Fuller, 2006; Crawford, 2017). These millets have been cultivated and harvested in many countries as food crops, especially in Asia and Africa (Anderson and Martin, 1949). Compared with the long domestication history of major crops that extends back to the early Holocene, the domestication or utilization history of other useful species is short or unclear (Zohary et al., 2012), and might be partially due to a lack of evidence. The development of new methods and proxies for the identification criteria of crop phytoliths has revealed the early domestication process of many species (Denham et al., 2003; Piperno and Stothert, 2003; Ezell et al., 2006; Horrocks and Rechtman, 2009; Piperno et al., 2009; Yang et al., 2013; Yang et al., 2015), and implies the possibility of using a similar method to investigate the early exploitation of Panicoideae species. Phytoliths are more stable under various preservation conditions and are generally abundant (Wang and Lu, 1993; Piperno, 2006). Moreover, inflorescences are the part of the plant generally collected for harvest; the occurrence of inflorescence phytoliths could reflect these activities. Thus, investigating inflorescence phytoliths in Panicoideae species could improve our understanding of the early process of plant resource exploitation.

In this study, we examined inflorescence phytoliths in every bract of a single specimen of the 38 most common Panicoideae species in China to provide a preliminary detailed phytolith morphology dataset. Further, we investigated the morphometric differences in a phytolith morphotype that we propose naming Interdigitating (see the results for a detailed description) on the lemma and palea of *Digitaria*, *Oplismenus*, and *Paspalum* genera to determine to what level (at genus, section, or species) the morphological traits might be robust. We also report on novel phytolith types that may be of high taxonomic value. This study provides insight into inflorescence phytoliths and reinforces the importance of treatment that preserves the anatomical position of phytolith in different bracts. Our detailed description of phytoliths in every bract of the inflorescence could provide the baseline information for further archaeological and taxonomical studies.

## Materials and Methods

### Sample Collection and Pretreatment

A total of 38 species (one specimen per species) (**Table 1**) were collected to investigate the morphological differences of phytoliths in the inflorescence of common Panicoideae plants in China. These species included the most common weeds and several minor crops from across China. They were collected during several field trips over decades led by colleagues from the Institute of Geology and Geophysics, the Chinese Academy of Sciences, and China Agricultural University, and identified by colleagues from the Institute of Botany, Chinese Academy of Sciences.

**Table 1:** Species involved in this study with their spikelet types and phytoliths types.

Tribe	Genus	Species	Spikelet types^1^	Phytoliths types^2^
Andropogoneae	*Apluda*	*Apluda mutica* L.	Type III	1-I
Andropogoneae	*Arthraxon*	*Arthraxon hispidus* (Trin.) Makino	Type III	1-II
Andropogoneae	*Bothriochloa*	*Bothriochloa ischcemum* (L.) Keng	Type III	1-III
Andropogoneae	*Capillipedium*	*Capillipedium assimile* (Steud.) A. Camus	Type III	1-IV
Andropogoneae	*Cymbopogon*	*Cymbopogon goeringii* (Steud.) A. Camus	Type I	1-V
Andropogoneae	*Eremopogon*	*Eremopogon delavayi* (Hack.) A. Camus	Type III	1-VI
Andropogoneae	*Coix*	*Coix lacryma-jobi* var. *ma-yuen* (Romanet du Caillaud) Stapf	Type II	2-I
Andropogoneae	*Coix*	*Coix lacryma-jobi* L.	Type II	2-II
Andropogoneae	*Eulalia*	*Eulalia speciosa* (Debeaux) Kuntze	Type III	3-I
Andropogoneae	*Hackelochloa*	*Hackelochloa granularis* (L.) Kuntze	Type II	3-II
Andropogoneae	*Imperata*	*Imperata cylindrica* (L.) P. Beauv.	Type III	3-III
Andropogoneae	*Ischaemum*	*Ischaemum anthephoroides* (Steud.) Miq.	Type III	3-IV
Andropogoneae	*Microstegium*	*Microstegium ciliatum* (Trin.) A. Camus	Type III	4-I
Andropogoneae	*Microstegium*	*Microstegium nudum* (Trin.) A. Camus	Type III	4-II
Andropogoneae	*Miscanthus*	*Miscanthus floridulus* (Labill.) Warb. ex K. Schum. & Lauterb.	Type III	4-III
Andropogoneae	*Miscanthus*	*Miscanthus nepalensis (Trinius) Hackel*	Type III	4-Iv
Andropogoneae	*Miscanthus*	*Miscanthus sinensis* Anderss.	Type III	4-V
Andropogoneae	*Saccharum*	*Saccharum arundinaceum* Retz.	Type III	5-I
Andropogoneae	*Saccharum*	*Saccharum rufipilum* Steudel	Type III	5-II
Andropogoneae	*Sorghum*	*Sorghum bicolor* (L.) Moench	Type II	5-III
Andropogoneae	*Spodiopogon*	*Spodiopogon sibiricus* Trin.	Type III	5-IV
Andropogoneae	*Spodiopogon*	*Spodiopogon tainanensis* Hayata	Type III	5-V
Andropogoneae	*Themeda*	*Themeda caudata* (Nees) A. Camus	Type I	6-I
Andropogoneae	*Themeda*	*Themeda japonica* (Willd.) Tanaka	Type I	6-II
Zeugiteae	*Lophatherum*	*Lophatherum gracile* Brongn.	Type III	6-III
Paniceae	*Digitaria*	*Digitaria chrysoblephara* Fig. & De Not.	Type IV	7-I
Paniceae	*Digitaria*	*Digitaria ciliaris* (Retz.) Koeler	Type IV	7-II
Paniceae	*Digitaria*	*Digitaria sanguinalis* (L.) Scop.	Type IV	7-III
Paniceae	*Digitaria*	*Digitaria ischaemum* (Schreb.) Schreb.	Type IV	7-IV
Paniceae	*Digitaria*	*Digitaria violascens* Link	Type IV	7-V
Paniceae	*Setaria*	*Setaria faberi* R.A.W. Herrm.	Type IV	8-I
Paniceae	*Setaria*	*Setaria pallidifusca* (Schumach.) Stapf et Hubb.	Type IV	8-II
Paniceae	*Setaria*	*Setaria plicata* (Lam.) T. Cooke	Type IV	8-III
Paniceae	*Setaria*	*Setaria pumila (Poiret) Roemer & Schultes*	Type IV	8-IV
Paniceae	*Oplismenus*	*Oplismenus undulatifolius* (Ard.) P. Beauv.	Type IV	9-I
Paniceae	*Oplismenus*	*Oplismenus compositus* (L.) P. Beauv.	Type IV	9-II
Paspaleae	*Paspalum*	*Paspalum orbiculare* G. Forst.	Type IV	9-III
Paspaleae	*Paspalum*	*Paspalum dilatatum* Poir.	Type IV	9-IV

^1^Spikelet types correspond to **Figure 1**.

^2^Details of phytoliths types in the inflorescence bracts were described in the supplementary file and shown in the **Supplementary Figures 1**–**9**.

**Figure 1:**
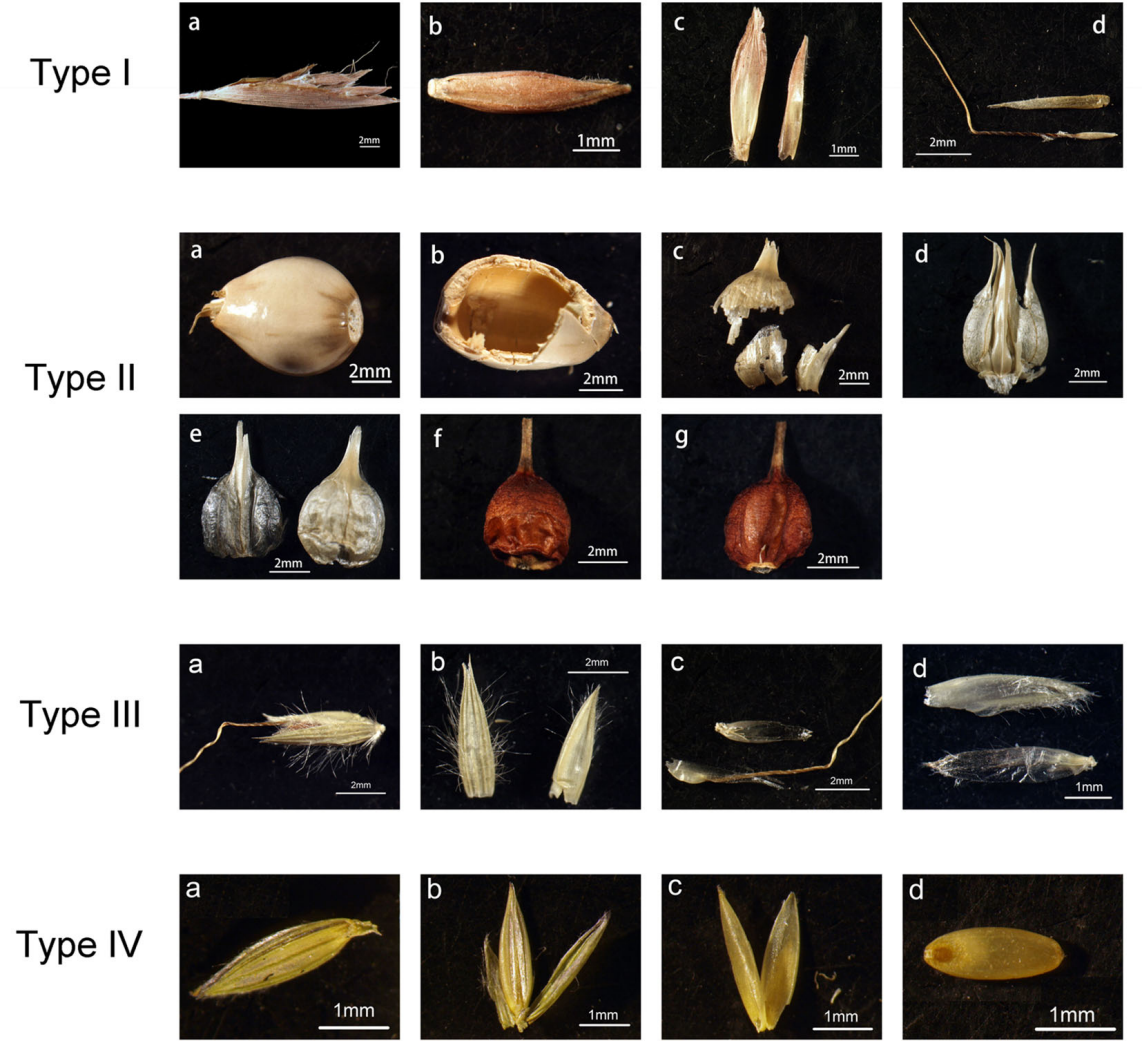
An illustration of dissected parts of spikelet for study. Type I to IV illustrate the different structures of spikelet in the samples, samples with the same type have similar structures. Type I: the spikelet of *Cymbopogon goeringii*, a one spikelet covered with the involucre, b an image of a floret, c the glumes, d the lemmas. Type II: the spikelet of *Coix lacryma-jobi*, a one spikelet covered with the involucre, b an image of the involucre, c and d the glumes, e the lemmas, f and g the seed. Type III: the spikelet of *Spodiopogon sibiricus*, a image of a spikelet, b the glumes, c the first lemma (lower one) and first palea (upper one), d the second lemma (upper one) and second palea (lower one). Type IV: the spikelet of *Digitaria sanguinalis*, a the image of a spikelet, b the lemma of sterile floret (middle one) and the glumes (left and right ones), c the lemma (left) and palea (right) of fertile floret, d the seed.

Mature spikelets from the inflorescence of collected samples (more than three entire spikelets from the same specimen) were dissected into different parts according to plant anatomy and included the following five parts: (1) involucre, (2) glume, (3) lemma, (4) palea, and (5) seed. All samples were divided into four groups (**Figure 1**) according to the dissection results: type I, with a thin and soft involucre covering other bracts and the seed; type II, with a thick and hard involucre or glume covering other bracts and the seed; type III, with thin and soft bracts; and type IV, with thicker and harder lemma and palea covering the seed compared to those of type III. After dissection under a microscope, every part (except the seed) was ultrasonically cleaned and dried for further treatment.

### Phytolith Preparation for *In Situ* Analysis

Whereas traditional wet oxidation methods for phytolith extraction (Piperno, 1988) can easily break down and disarticulate phytoliths (Jenkins, 2009), we prepared our samples following the published methods of Lu et al., 2009b, with minor modifications, to ensure that the phytoliths in the whole bract structures remained articulated and undamaged. For the bracts that were thick and hard (e.g. involucre from type II, or lemma and palea from type IV), a saturated nitric acid (HNO_3_) treatment was used. A total of 5–10 ml HNO_3_ was added to each bract (to merge the bracts) in a 15 ml centrifuge tube, then the sample was placed in a water bath at 50–60 ºC. When the bract turned transparent, all contents in the tube were poured into a glass dish, and the bract was carefully moved onto a slide. Distilled water was used to wash the bract on the slide to remove the HNO_3_, and absolute ethanol was used to wash the bract to remove the water. After the bract was dry, a drop of xylol was added to it. Before the xylol was totally volatilized, a drop of Canada Balsam was added, and the bract was covered with a cover glass. All procedures were performed in a fume cupboard. Similar procedures were followed for bracts that were thin and soft, except saturated nitric acid was replaced with hydrogen peroxide (H_2_O_2_). This method increased the chances of keeping the whole bract structure undamaged and allowed for articulated or *in situ* observation of the phytoliths in the bracts. At least two replicates were prepared for each bract.

### Interdigitating Phytolith Measurement

**Figure 2:**
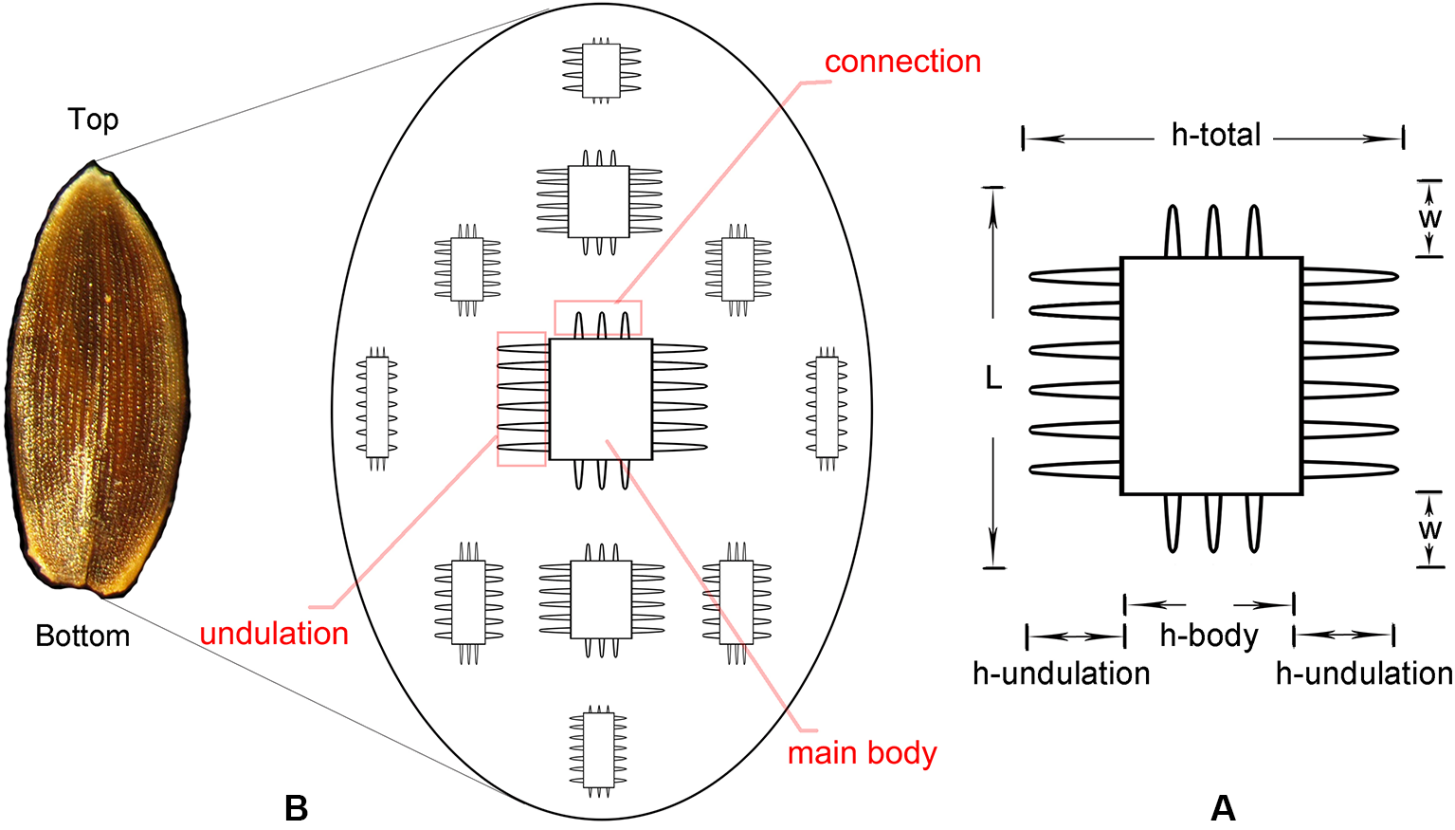
Conceptual sketch of the distribution of Interdigitating phytolith **(B)** and parameters used to describe the morphology of Interdigitating phytolith **(A)**.

The phytolith morphotype that we name Interdigitating has been reported to be a useful tool in discriminating samples at the genus level for some taxa (Lu et al., 2009b; Madella et al., 2013; Weisskopf and Lee, 2016; Ge et al., 2018), however, discrimination at species level requires the assistance of morphometric analysis (Zhang et al., 2011; Zhang et al., 2018). The Interdigitating phytoliths from *Digitaria*, *Paspalum*, and *Oplismenus* genera were employed to aid in morphometric discrimination from each other in our samples. The measurement parameters are shown in **Figure 2A**, and are described as follows: h-total is the width of the whole undulation pattern; h-undulation is the mean value of the two individual undulation parts; h-body is the difference between h-total and h-undulation; w is the length of the protuberant ends, and was measured along one direction (either upward or downward) in the same sample; and L is the total length of the undulation patterns. Two additional parameters used were: (1) R (w/hu) = w/h-undulation; (2) R (hu/hb) = h-undulation/h-body. All parameters were measured in 150 individuals [the number has been tested by the suggested formula (Ball et al., 2016b)] of each species and were measured in different areas. Fifty measurements were taken near the base area, 50 from the center area, and 50 from the top area. Data parameters are shown in **Table 2**. All observations and measurements of inflorescence phytolith parameters were conducted under a Leica DM 750 microscope with 400× magnification. Statistical analysis (conical discriminate analysis) was performed using IBM SPSS Statistics 24 software.

**Table 2:** Parameters of the Interdigitating phytolith in *Digitaria*, *Paspalum*, and *Oplismenus*.

Species	Location	Statistics	Parameters (μm)						
			w	L	h-Undulation	h-Body	h-Total	R（w/hu）	R（hu/hb）
*D. sanguinalis*	Base	Mean	1.70	16.07	9.57	3.16	22.29	0.18	3.38
		Median	1.63	14.70	9.05	3.06	20.55	0.19	3.16
		SD	0.07	0.61	0.29	0.18	0.69	0.01	0.16
	Center	Mean	2.22	25.49	21.93	9.92	53.78	0.10	2.33
		Median	2.11	25.89	21.98	9.80	53.57	0.10	2.25
		SD	0.09	0.71	0.29	0.27	0.51	0.00	0.09
	Top	Mean	2.08	26.40	7.53	7.05	22.11	0.33	1.12
		Median	2.04	26.24	6.49	7.03	19.92	0.26	0.92
		SD	0.08	0.96	0.45	0.22	0.94	0.03	0.08
*D. chrysoblephara*	Base	Mean	2.41	26.98	9.75	8.01	27.52	0.27	1.46
		Median	2.44	26.33	9.73	8.27	27.99	0.25	1.30
		SD	0.09	1.00	0.48	0.39	0.93	0.01	0.14
	Center	Mean	3.48	29.78	20.61	8.49	49.71	0.17	2.68
		Median	3.59	31.05	20.28	8.17	49.33	0.17	2.59
		SD	0.12	0.78	0.33	0.35	0.74	0.01	0.16
	Top	Mean	2.46	25.30	9.12	7.29	25.54	0.30	1.34
		Median	2.29	24.02	9.59	7.46	25.96	0.26	1.31
		SD	0.09	0.88	0.40	0.24	0.84	0.02	0.08
*D. ciliaris*	Base	Mean	2.26	26.38	9.84	6.64	26.31	0.26	1.62
		Median	2.08	26.61	10.21	6.25	27.36	0.22	1.50
		SD	0.11	0.92	0.41	0.29	0.90	0.02	0.10
	Center	Mean	3.11	27.43	20.13	8.07	48.33	0.15	2.64
		Median	3.10	27.21	20.32	7.67	48.68	0.16	2.62
		SD	0.10	0.74	0.22	0.28	0.43	0.00	0.09
	Top	Mean	3.01	29.41	9.36	6.57	25.30	0.34	1.52
		Median	2.95	29.36	9.06	6.55	24.99	0.32	1.38
		SD	0.12	0.86	0.33	0.22	0.69	0.02	0.08
*P. orbiculare*	Base	Mean	4.43	39.75	9.98	17.03	36.99	0.47	0.60
		Median	4.27	36.57	9.57	15.86	36.95	0.40	0.59
		SD	0.17	1.92	0.35	0.59	1.14	0.03	0.02
	Center	Mean	4.72	59.98	19.44	27.25	66.13	0.25	0.75
		Median	4.88	59.27	19.38	26.59	65.69	0.25	0.69
		SD	0.17	1.53	0.38	0.73	0.89	0.01	0.03
	Top	Mean	5.32	51.37	11.56	15.12	38.24	0.48	0.79
		Median	5.04	48.71	11.48	14.60	37.43	0.43	0.77
		SD	0.23	2.38	0.37	0.60	1.22	0.03	0.03
*P. dilatatum*	Base	Mean	7.46	59.75	9.00	12.26	30.26	0.86	0.75
		Median	7.65	60.10	9.40	11.72	30.99	0.82	0.70
		SD	0.27	2.05	0.31	0.42	0.89	0.04	0.03
	Center	Mean	5.61	44.78	14.72	18.48	47.91	0.40	0.81
		Median	4.98	45.57	14.85	18.13	47.15	0.33	0.80
		SD	0.32	1.32	0.30	0.48	0.78	0.03	0.03
	Top	Mean	3.67	40.76	8.28	11.24	27.79	0.50	0.76
		Median	3.21	39.32	7.94	10.85	25.82	0.45	0.82
		SD	0.20	1.20	0.40	0.45	1.11	0.04	0.03
*O. compositus*	Base	Mean	1.47	85.83	9.05	6.70	24.81	0.19	1.43
		Median	1.19	86.19	9.11	5.95	24.10	0.18	1.33
		SD	0.11	2.78	0.45	0.34	1.11	0.02	0.08
	Center	Mean	1.31	62.66	19.17	9.57	47.90	0.07	2.06
		Median	1.21	61.43	19.41	9.32	48.12	0.06	2.12
		SD	0.06	2.03	0.28	0.31	0.63	0.00	0.08
	Top	Mean	1.72	90.69	10.06	6.65	26.77	0.19	1.66
		Median	1.37	87.37	10.24	6.36	25.99	0.15	1.78
		SD	0.14	3.03	0.44	0.38	0.97	0.02	0.09
*O. undulatifolius*	Base	Mean	2.35	83.02	9.96	5.66	25.59	0.25	1.85
		Median	1.79	82.30	9.31	5.34	24.51	0.18	1.83
		SD	0.22	3.84	0.35	0.22	0.81	0.02	0.08
	Center	Mean	2.84	74.28	17.82	8.47	44.11	0.16	2.17
		Median	1.79	74.41	17.62	8.29	43.57	0.11	2.14
		SD	0.36	2.71	0.29	0.25	0.70	0.02	0.06
	Top	Mean	2.57	93.30	10.92	5.82	27.67	0.25	2.00
		Median	1.93	90.85	11.65	5.51	28.47	0.18	1.90
		SD	0.23	3.73	0.28	0.21	0.59	0.03	0.09

SD, standard deviation.

## Results

In general, phytoliths were abundant in the inflorescence bracts of the studied species. The distribution of phytolith types in the inflorescence bracts are shown in **Table 3**, detailed descriptions can be found in the Supplementary file, and the details of phytolith morphology in different inflorescence bracts can be found in **Supplementary Figures 1**–**9**.

**Table 3:** Phytoliths types in different bracts of the studied samples.

Species	Supplementary Figure	Phytolith types					
		involucre	glume	lemma		palea	
				fertile/upper floret	sterile/lower floret	fertile/upper floret	sterile/lower floret
*Apluda mutica*	1-I	NB	BIL conc var1 POL conc var1 ACU INT nPAP nun acon rbod	ACU	NB	NP	NB
*Arthraxon hispidus*	1-II	NB	BIL conv vars BIL conc var2 INT sPAP nun acon rbod ACU	BIL conv var2, INT sPAP nun acon rbod ACU	NB	NP	NB
*Bothriochloa ischcemum*	1-III	NB	BIL conc var1 BIL conc var2 INT nPAP nun acon rbod ACU	BIL conv var1 ACU	NB	NP	NB
*Capillipedium assimile*	1-IV	NB	BIL conc var1 POL conc var1 ELO_DET/DEN ACU	NP	NB	NP	NB
*Cymbopogon goeringii*	1-V	BIL conv var5/6 BIL conv vars ELO_DEN/DET ACU	BIL conv var5/6 BIL conv vars ELO_DEN/DET ACU	ELO_ENT ELO_DEN/DET ACU	ELO_ENT ELO_DEN/DET ACU	NB	NB
*Eremopogon delavayi*	1-VI	NB	BIL conv var1 ACU_BUL ELO_DEN/DET ACU	ACU_BUL	NP	NB	NB
*Coix lacryma-jobi* var. *ma-yuen*	2-I	BIL conv var1 BIL conv var 5/6 CRO conc var1 POL conc var1 ELO_DEN/DET SREC_DET	BIL conc var1 CRO conc var1 POL conc var1 ELO_DEN/DET pREC_DET ACU	BIL conc var1 CRO conc var1 POL conc var1 pREC_DET ACU	BIL conc var1 CRO conc var1 POL conc var1 pREC_DET ACU	BIL conc var1 CRO conc var1	NB
*Coix lacryma-jobi*	2-II	BIL conc var1 CRO conc var1 POL conc var1 BLO_AMO	BIL conc var1 CRO conc var1 POL conc var1 ELO_DEN/DET pREC_DET ACU	BIL conc var1 CRO conc var1 POL conc var1 ACU	BIL conc var1 CRO conc var1 POL conc var1 ACU	NB	NB
*Eulalia speciosa*	3-I	NB	BIL conv vars ELO_DEN/DET ACU	BIL conv vars ELO_DEN/DET ACU	NP	NP	NP
*Hackelochloa granularis*	3-II	NB	INT sPAP nun acon rbod BIL conc var1 ACU ACU_BUL	NP	NP	NP	NP
*Imperata cylindrica*	3-III	NB	BIL conv vars ACU	BIL conv vars ACU	NP	NP	NB
*Ischaemum anthephoroides*	3-IV	NB	RON BIL conv vars ACU	BIL conv var1 ACU	BIL conv var1 ELO_DEN/DET ACU	BIL conv var1	ELO_DEN/DET
*Microstegium ciliatum*	4-I	NB	BIL conv vars ACU	BIL conv vars ELO_DEN/DET ACU	NP	NB	NB
*Microstegium nudum*	4-II	NB	BIL conv vars ELO_DEN/DET ACU	ACU ACU_BUL	NP	NB	NB
*Miscanthus floridulus*	4-III	NB	BIL conv vars ACU	BIL conv vars	NP	NB	NB
*Miscanthus nepalensis*	4-Iv	NB	BIL conv vars ELO_DEN/DET ACU_BUL ACU	BIL conv var1 ELO_DET_CYL ACU	NP	NB	NB
*Miscanthus sinensis*	4-V	NB	BIL conv vars ELO_DEN/DET ELO_ENT ACU ACU_BUL	BIL conv vars ELO_DEN/DET ELO_ENT ACU ACU_BUL	BIL conv vars ELO_DEN/DET ELO_ENT ACU ACU_BUL	NP	NB
*Saccharum arundinaceum*	5-I	NB	BIL conv vars ELO_DEN/DET ACU	BIL conc var1 POL conc var1 ACU	BIL conc var1 POL conc var1 ACU	NP	NB
*Saccharum rufipilum*	5-II	NB	BIL conv vars ELO_DEN/DET ACU	ELO_DEN/DET ACU ACU_BUL	ELO_DEN/DET ACU ACU_BUL	NB	NB
*Sorghum bicolor*	5-III	NB	BIL conv var1 BIL conv vars BIL conv var5/6 ELO_DEN/DET ELO_DEN tent ACU	BIL conv vars ACU	BIL conv vars ACU	NB	NB
*Spodiopogon sibiricus*	5-IV	NB	BIL conc var5/6 BIL conc vars BIL conv var 5/6 BIL conv vars ACU	BIL conc vars	NP	NP	NP
*Spodiopogon tainanensis*	5-V	NB	BIL conc var 5/6 BIL conc vars ACU ELO_DEN/DET	NP	NP	NP	NP
*Themeda caudata*	6-I	BIL conv vars BIL conv var7 ACU	BIL conv vars BIL conv var7 ACU	BIL conv var1 ACU	NP	NP	NP
*Themeda japonica*	6-II	BIL conv var 7 ELO_DEN/DET ELO_ENT_CYL ACU ELO_PAR	BIL conv var 7 ELO_DEN/DET ELO_ENT_CYL ACU ELO_PAR PRI	NP	NP	NP	NP
*Lophatherum gracile*	6-III	NB	BIL conv var1 BIL conv vars ACU ACU_BUL	BIL conv var1 BIL conv vars ACU	BIL conv var1 BIL conv vars ACU	BIL conv var1	NB
*Digitaria chrysoblephara*	7-I	NB	BIL conc var1 ELO_DEN/DET ACU	INT mPAP sun acon rbod	BIL conv var1 ELO_DEN/DET ACU	INT mPAP sun acon rbod	NB
*Digitaria ciliaris*	7-II	NB	BIL conc var1 ELO_DEN/DET ACU	INT mPAP sun acon rbod	BIL conv var1 ELO_DEN/DET ACU	INT mPAP sun acon rbod	NB
*Digitaria sanguinalis*	7-III	NB	BIL conc var1 ELO_DEN/DET ACU	INT mPAP sun acon rbod	BIL conv var1 ELO_DEN/DET ACU	INT mPAP sun acon rbod	NB
*Digitaria ischaemum*	7-IV	NB	BIL conv var1 BIL conv vars POL conv var1 ELO_DEN/DET ACU	dPAP	BIL conv var1 BIL conv vars POL conv var1 ELO_DEN/DET ACU	dPAP	NB
*Digitaria violascens*	7-V	NB	BIL conv var1 BIL conv vars POL conv var1 ELO_DEN/DET ACU	dPAP	BIL conv var1 BIL conv vars POL conv var1 ELO_DEN/DET ACU	dPAP	NB
*Setaria faberi*	8-I	NB	BIL conc var1 ACU	INT mPAP oun scon rbod	BIL conc var1 ACU	INT mPAP oun scon rbod	NB
*Setaria pallidifusca*	8-II	NB	BIL conv var1 ACU	INT mPAP oun scon rbod	BIL conv var1 ACU	INT mPAP oun scon rbod	NB
*Setaria plicata*	8-III	NB	BIL conc var1 ACU	INT mPAP oun scon rbod	BIL conc var1 ACU	INT mPAP oun scon rbod	NB
*Setaria pumila*	8-IV	NB	BIL conv var1 ACU	INT mPAP oun scon rbod	BIL conv var1 ACU	INT mPAP oun scon rbod	NB
*Oplismenus undulatifolius*	9-I	NB	BIL conc var1 CRO conc var1 CRO conc var5/6 POL conc var5/6 ELO_DEN/DET INT nPAP nun scon rbod ACU	INT nPAP oun acon rbod	CRO conc var1 POL conc var1 ELO_DEN/DET INT nPAP nun acon rbod ACU	INT nPAP oun scon rbod	CRO conc var1 POL conc var1 ELO_DEN/DET INT nPAP nun scon rbod ACU
*Oplismenus compositus*	9-II	NB	BIL conc var1 CRO conc var1 POL conc var1	INT nPAP sun scon rbod	BIL conc var1 CRO conc var1 POL conc var1	INT nPAP sun scon rbod	NB
*Paspalum orbiculare*	9-III	NB	BIL conc var1 POL conc var1 ACU	BIL conc var2 CRO conc var2 POL conv var2 INT mPAP oun acon obod	BIL conc var1 POL conc var1 ACU	BIL conc var2 CRO conc var2 POL conv var2 INT mPAP oun acon obod	NB
*Paspalum dilatatum*	9-IV	NB	BIL conc var1 POL conc var1 ACU	BIL conc var2 CRO conc var2 POL conv var2 INT mPAP oun acon rbod	BIL conc var1 POL conc var1 ACU	BIL conc var2 CRO conc var2 POL conv var2 INT mPAP oun acon rbod	NB

The abbreviations used in the table refer to 1) NB, no bract; NP, no phytolith observed; 2) BIL, Bilobate; CRO, Cross; POL, Polylobate; conv, convex ends; conc, concave ends; var1, variant 1; var2, variant 2; var5/6, variant 5/6; var7, variant 7; vars, variant saddle-like; 3) ELO, Elongate; DET, dentate; CYL, cylindric; ENT, entire; DEN, dendritic; tent, multi tent-like arch top; PAR, Papillar; 4) INT, Interdigitating; nPAP, no Papillate; mPAP, Papillate attached with main body; sPAP, Papillate separated with main body; sun, smooth-type undulation; nun, n-type undulation; oun, Ω-type undulation; scon, smooth connection; acon, articulated connection; obod, ovate main body; rbod, rectangular main body; 5) RON, Rondel with small chamber; ACU, Acute; ACU_BUL, Acute bulbosus; dPAP, disaggregated Papillate; BLO_AMO, Blocky amoeboid; sREC_DET, Rectangular dentate with smooth short sides; pREC_DET, Rectangular dentate with protuberant short sides; PRI, Prismatic silicified hair base.

### Phytolith Nomenclature and Classification

**Figure 3:**
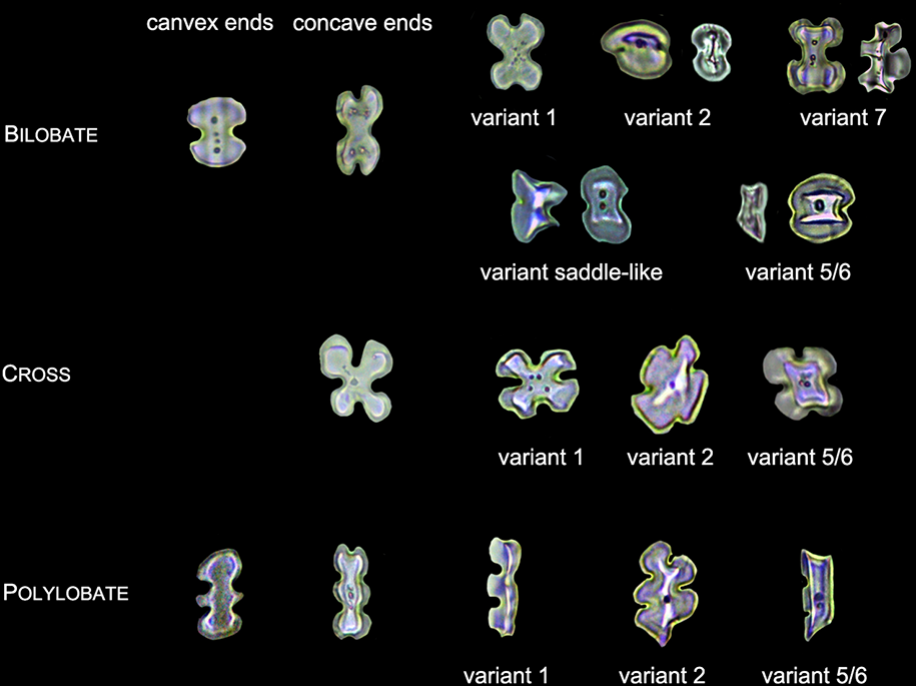
Illustration on the morphology of the Lobate phytoliths.

**Figure 4:**
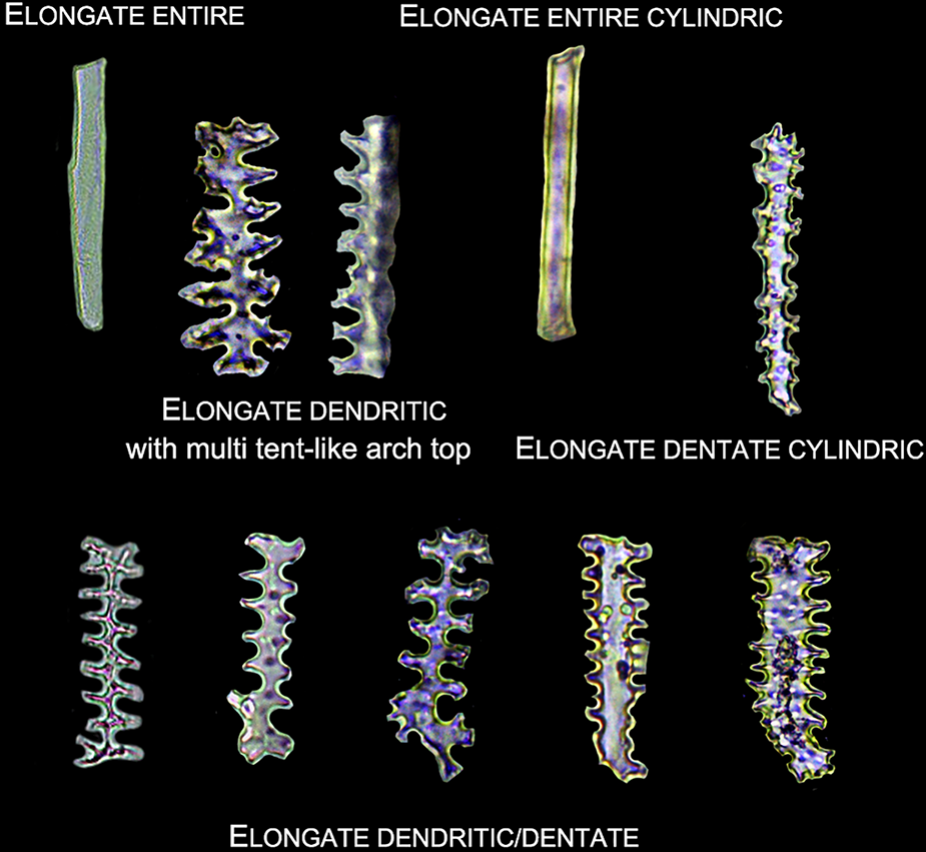
Illustration on the morphology of the Elongate phytoliths.

**Figure 5:**
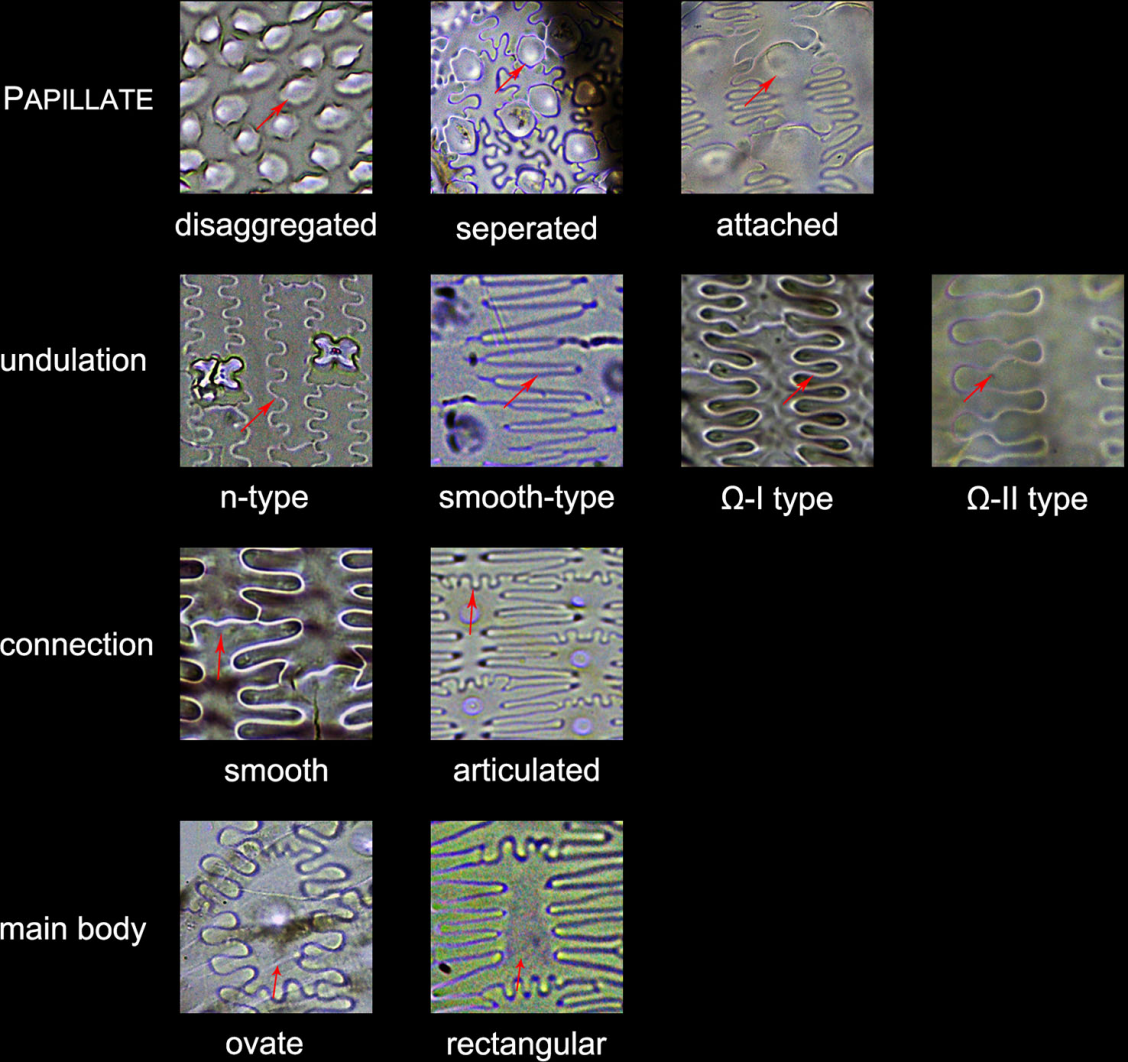
Illustration on the morphological traits of the Interdigitating phytolith. Parts of the Interdigitating phytolith refer to **Figure 2B**.

**Figure 6:**
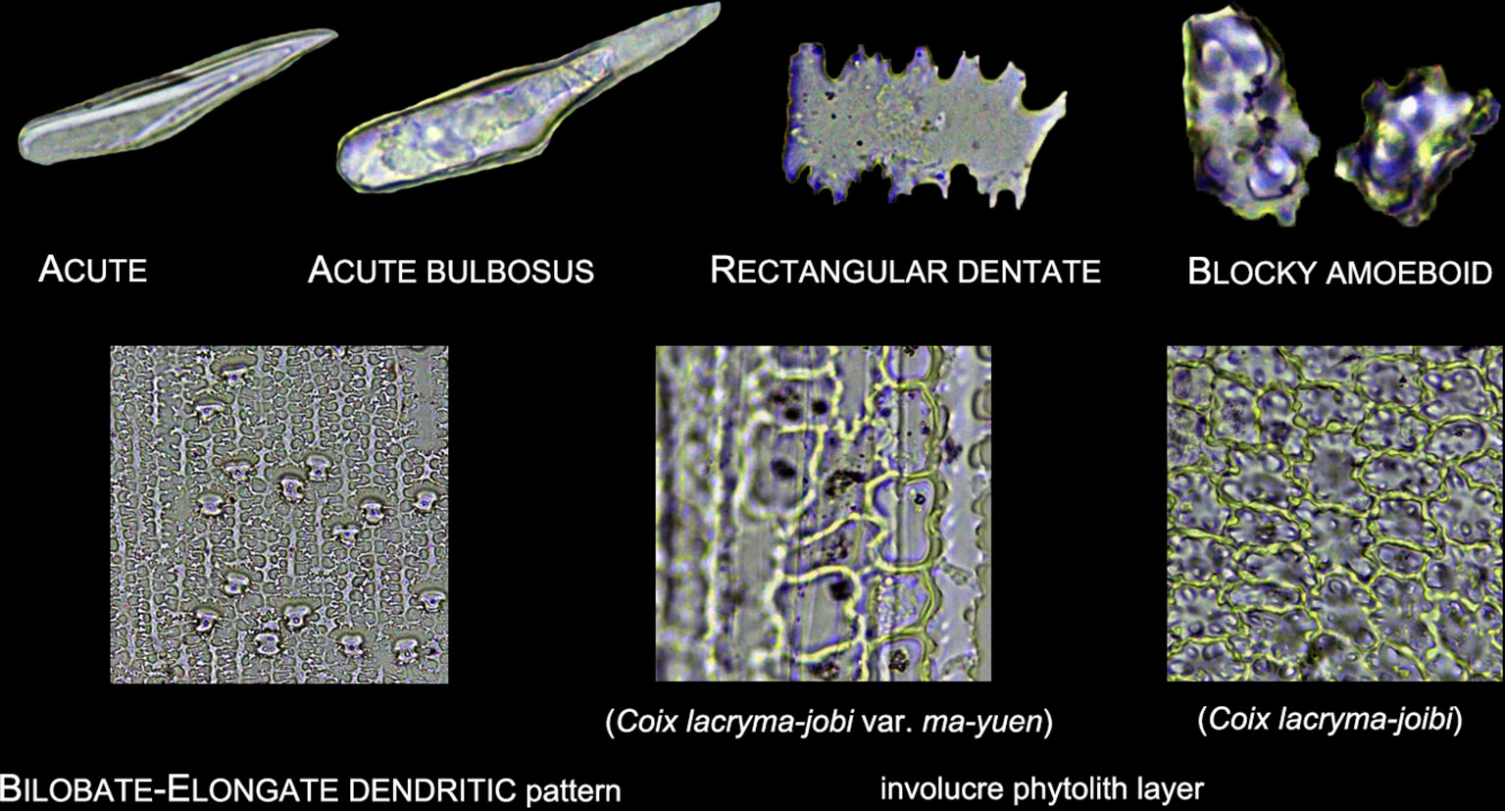
Illustration on the morphology of other phytoliths types.

Phytolith morphology nomenclature followed ICPN 2.0 rules (ICPT, 2019), and the description and classification of phytolith morphology followed those of previous studies:

1. Lobate phytoliths were firstly classified into Bilobate, Cross, and Polylobate, then classified by convex and concave ends (Lu and Liu, 2003; Radomski and Neumann, 2011), and finally classified by the 3-D structure into variant 1 (identical top and base), variant 2 (tent-like arch top), variant 5/6 (trapezoidal or rectangular structure on the top), variant 7 (bilobate base and cross top), and variant saddle-like (saddle-like top with bilobate base) (Piperno, 1984; Radomski and Neumann, 2011). Different types of Lobate phytoliths are shown in **Figure 3**.
2. Elongate phytoliths were classified into Elongate entire, Elongate dendritic/dentate, Elongate entire cylindric, Elongate dentate cylindric (the Elongate entire cylindric and Elongate dentate cylindric have an obvious cylindrical rod body while the other Elongate types have a relatively flat plate body under microscope), and Elongate phytoliths with a special 3-D morphology were also observed in some specimens; the 3-D morphology was described following previously published methods (Piperno and Pearsall, 1993): first, according to the morphology of the base (e.g. Elongate dendritic/dentate base), then according to the morphology of the top (e.g. multi-tent-like echinate arch top). The presence of Elongate dendritic/dentate phytoliths have been extensively reported in the inflorescence of cereals (Parry and Smithson, 1966; Hodson and Sangster, 1988; Rosen and Weiner, 1994; Ball et al., 2009), and have been recognized as a “silica skeleton” (Rosen, 1992; Madella et al., 2014). However, the “silica skeleton” has been previously used not only to refer to Elongate dendritic layers derived from silicified lumen of epidermal long cells, but also to the silica layer between the epidermal cuticle layer and the epidermal cells (Rosen, 1992; Madella et al., 2013; Madella et al., 2014; Weisskopf and Lee, 2016; Ge et al., 2018). Thus, in this paper to clarify the different morphology and anatomical origins, the ‘Elongate dendritic’ only refers to the phytoliths derived from epidermal long cells, and the “Interdigitating” only refers to the phytoliths derived from the silica layer between the epidermal cuticle layer and the epidermal cells. Different types of Elongate phytoliths are shown in **Figure 4**.
3. Other morphotypes included Acute, Acute bulbosus, Papillate, Blocky amoeboid, and Rectangular dentate. Acute we distinguish as being derived from the silicified hair cell wall, while Acute bulbosus from the entire silicified hair cell. Papillate we classified into disaggregated Papillate and silica layer Papillate (Papillate on the silica layer). Further, the silica layer Papillate we divided into Papillate attached to the main body and Papillate separated from the main body (**Figure 5**). Blocky amoeboid was a novel phytolith type reported in this study; the morphology includes a rounded rectangle/oblong base and a semi-cubic/globular top, with an irregular granulated surface. Blocky amoeboid phytoliths are derived from the silicified epidermal cells of the involucre, which could aggregate together to form a silica cell layer covering the surface of the involucre. Rectangular dentate phytoliths were also a novel phytolith type observed in this study; the morphology includes a tabular/rectangle main body and a ruminate pattern along the two long sides that could be further divided into smooth ends (the short sides) and protuberant ends. Morphology of these phytolith types are shown in **Figure 6**.
4. The new morphotype, Interdigitating, that we propose in this manuscript, consisted of a silica layer between the epidermal cells and the epidermal cuticle layer. This type was named after Parry and Hodson’s first observation of the morphotype in *S. italica*, in which they described what they observed on inflorescence bracts as “interdigitating epidermal cells” (Parry and Hodson, 1982); however, these could be the silica layer covering the surface of the lemma and palea. Other names that have been used by other studies to describe this type of phytolith include “silica skeleton” (Rosen, 1992; Madella et al., 2013; Madella et al., 2014; Weisskopf and Lee, 2016), “dendriform” (Lu et al., 2005), “silicified epidermal long cells” (Lu et al., 2009a; Lu et al., 2009b; Zhang et al., 2011; Kealhofer et al., 2015), and “epidermal silica layer” (Ge et al., 2018). As the anatomical origin of this type of phytolith has been discussed by Ge (Ge et al., 2018), and associated with a study on rice husk (Yoshida et al., 1962), we propose Interdigitating as the formal name for this phytolith type to show its different anatomical origin and morphology. Previous studies have shown that morphological trait combinations could be helpful in discriminating Interdigitating. In the present study, we followed the description of *Setaria*, *Panicum* (Lu et al., 2009b), and *Echinochloa* (Ge et al., 2018) and used the morphological traits of Papillate (present or not), undulation patterns (n-type, smooth-type, and Ω-type), ending structure (smooth connection or articulated connection), and main body (ovate or rectangular) to describe Interdigitating morphology. All the traits that describe Interdigitating morphology and are shown in **Figure 5**.
5. As the treatment retained the undamaged anatomical structure, the different types of phytoliths could combine to form a pattern of taxonomic value. In this study, one pattern, a Bilobate- Elongate dendritic/dentate pattern, and a special involucre phytolith layer were observed (**Figure 6**). The Bilobate-Elongate dendritic/dentate pattern was comprised of Bilobate and Elongate dendritic/dentate, one Bilobate and one Elongate dendritic/dentate alternating formed the common pattern. Their long axes were parallel to the veins, sometimes Bilobate was replaced by Papillate or Acute or just disappeared. A similar pattern has been reported in the inflorescence of cereals, the Papillate- Elongate dendritic/dentate pattern (Parry and Smithson, 1966; Rosen, 1992; Tubb et al., 1993), which includes a combination of Papillate and Elongate dendritic/dentate. Sometimes Papillate could be replaced by the Rondel, and the Papillate in this pattern has pits (radiating marks) on the base. Thus, the Bilobate- Elongate dendritic/dentate pattern could be a potential tool to distinguish between some Panicoideae grasses and Pooideae cereals. The involucre phytolith layer was found in *Coix lacryma-jobi* and *C. lacryma-jobi* var. *ma-yuen* on the surface of the involucre and was comprised of tightly connected phytoliths with various morphologies (could be stretched or condensed in morphology). This phytolith layer was reported for the first time in this study and recognized as the involucre phytolith layer. The involucre phytolith layer (**Figure 6**) differed among species in our samples: (1) In cultivated *C. lacryma-jobi* var. *ma-yuen*, the involucre phytolith layer was comprised of different types of phytoliths, including Bilobate (some could be condensed or stretched in morphology) and Elongate dendritic/dentate, each in a different column and with their long axis parallel to the veins; (2) In wild type *C. lacryma-jobi*, the involucre phytolith layer was only comprised of Blocky amoeboid, this type of phytolith was cubic or oblong with granules on the surface. They were tightly connected with each other and the column was parallel to the veins.

### Phytoliths in Different Inflorescence Bracts

Involucres were found in *Cymbopogon goeringii*, *C. lacryma-jobi* var. *ma-yuen*, *C. lacryma-jobi*, *Themeda caudata*, and *Themeda japonica*. In our samples phytoliths in the involucre could be divided into two groups, corresponding to spikelet type I and type II (**Figure 1**). In the involucres of *C. goeringii* (**Supplementary Figure 1**-**V**), *T. caudata* (**Supplementary Figure 6-I**), and *T. japonica* (**Supplementary Figure 6**-**II**), which were spikelet type I, phytolith types were mainly Bilobate, Elongate, and Acute (**Table 3**), and the phytoliths were separated from each other and presented a scattered distribution in the involucre. However, in the involucres of *C. lacryma-jobi* var. *ma-yuen* (**Supplementary Figure 2**-**I**) and *C. lacryma-jobi* (**Supplementary Figure 2**-**II**), which were spikelet type II, phytoliths were all tightly connected to form an involucre phytolith layer on the surface. The phytolith types were Lobate, Elongate, Rectangular dentate, and Blocky amoeboid.

Glumes were found in all studied species. Most of the glumes were thin and soft in the studied species; however, in *Sorghum bicolor* and *Hackelochloa granularis* (both are spikelet type II), glumes were thick and hard (*H. granularis* had a thin but hard glume). Thus, the phytolith type in the glumes appeared to differ: in thin and soft glumes, phytoliths were Lobate, Elongate, Elongate dendritic/dentate, and Acute, and scattered from each other; while in *S. bicolor* (**Supplementary Figure 5**-**III**), phytoliths were Lobate, Elongate, Elongate dendritic/dentate, and Acute, and the Bilobate- Elongate dendritic/dentate pattern covered most of the area of the glume surface. In the lower glume, Elongate dendritic with multi tent-like arch tops (**Figure 4**) were observed, and this type was only observed in the lower glume of *S. bicolor*. In *H. granularis* (**Supplementary Figure 3**-**II**), phytoliths in the glumes formed an Interdigitating with Papillate pattern separated from the main body, n-type undulation, articulated connection, and rectangle main body; this type of phytolith covered the entire surface of the glumes, and was the only type observed in *H. granularis*. An exception of the soft and thin glumes was *Arthraxon hispidus*, which produced weakly silicified Interdigitating (**Supplementary Figure 2**-**II**) and Bilobate phytoliths could be found among the Interdigitating.

Lemmas were found in most of the studied species and could be divided into lemmas of the sterile floret and lemmas of the fertile floret. Sterile lemmas were remarkably similar to the thin and soft glumes in morphology, and the phytolith types were the same. The fertile lemmas could be divided into two groups according to spikelet type (III and IV): fertile spikelet type III lemmas were similar to the glumes and sterile lemmas in morphology and the phytolith types were the same. Fertile spikelet type IV lemmas were hard and glossy, Interdigitating was the major phytolith type that covered the entire surface of the lemmas.

Paleas were similar to the lemmas; however, the paleas may stop growing or be absorbed during the growth of the inflorescence and finally disappear in some of the studied species. Phytoliths in the paleas could be the same as that in the lemmas. In the sterile paleas and fertile spikelet type III paleas, phytoliths were Lobate, Elongate, and Acute, and far fewer occurred compared to the number in the lemmas of the same floret. In the fertile spikelet type IV paleas, phytoliths formed the Interdigitating type, the same as those in the lemmas from the same floret.

In all the inflorescence bracts, Lobate, Elongate, Elongate dendritic/dentate, and Acute phytoliths were the most commonly observed phytolith types. The morphology of these types could overlap among different species and were of relatively low taxonomic value in distinguishing among the studied species. However, phytolith types from spikelet type II and IV (which usually produce more phytoliths than spikelet type I and III), namely the Blocky amoeboid, Rectangular dentate, Elongate dendritic with multi tent-like arch top, and Interdigitating, as well as the combination of phytolith types such as the Bilobate- Elongate dendritic/dentate pattern and the involucre phytolith layer showed higher taxonomic value in distinguishing among our samples of the studied species. The morphological traits are summarized in **Table 4**.

**Table 4:** Morphological traits of the inflorescence-types of phytoliths and the corresponding species.

Phytoliths types	Morphological traits *	Corresponding species
**BILOBATE-ELONGATE DENTATE/DENDRITIC pattern**	1 ELONGATE DENTATE/DENDRITIC separated by BILOBATE	*Cymbopogon goeringii, Eulalia speciosa, Microstegium nudum, Miscanthus floridulus, Miscanthus nepalensis, Miscanthus sinensis, Saccharum arundinaceum, Saccharum rufipilum, Sorghum bicolor*
	2 some BILOBATE replaced by ACUTE	*Eremopogon delavay*i
**ELONGATE DENDRITIC 3D**	1 ELONGATE DENDRITIC base 2 multi-tent like arch top	
	3 small pricks on the arch top	*Sorghum bicolor*
**INTERDIGITATING**	1.1 PAPILLATE separated with main body	*Arthraxon hispidus, Hackelochloa granularis*
	1.2 PAPILLATE attached to the main body 2.1 undulation smooth 3.1 connection articulated	
	4.1 main body rectangular	*Digitaria chrysoblephara, Digitaria ciliaris, Digitaria sanguinalis*
	2.2 undulation Ω-type 3.2 connection smooth
	4.1 main body rectangular	*Setaria faberi, Setaria pallidifusca, Setaria plicata, Setaria pumila*,
	3.1 connection articulated	
	4.1 main body rectangular	*Paspalum dilatatum*
	4.2 main body ovate	*Paspalum orbiculare*
	1.3 no PAPILLATE attached to the main body 2.1 undulation smooth 3.2 connection smooth	
	4.1 main body rectangular	*Oplismenus compositus*
	2.2 undulation Ω-type 3.1 connection articulated	
	4.1 main body rectangular	*Oplismenus undulatifolius*,
**BLOCKY AMOEBOID**	1 rounded rectangular or oblong base 2 semi-cubic or globular top	
	3 irregular granulated surface	*Coix lacryma-jobi*
**RECTANGULAR**	1 tabular main body	
**DENTATE**	2 ruminate pattern along the two long sides 3.1 smooth short sides	
	4.1 smooth surface	*Coix lacryma-jobi var. ma-yuen*
	3.2 protuberant short sides 4.2 granulated surface	*Coix lacryma-jobi*
**involucre phytoliths layer**	1.1 composed of BILOBATE and ELONGATE	*Coix lacryma-jobi var. ma-yuen*
	1.2 composed of BLOCKY AMOEBOI	*Coix lacryma-jobi*

The numbers before the descriptions showed the categories of morphological traits, using the combination of morphological traits from different categories could conduct identification of certain species.

### Morphological and Morphometric Approaches to the Interdigitating Phytolith

Among the phytolith types found in inflorescences with potentially high taxonomic value, the Interdigitating had more complex morphological traits than the others; it could be observed in *A. hispidus*, *H. granularis*, *Digitaria chrysoblephara*, *Digitaria ciliaris*, *Digitaria sanguinalis*, *Setaria faberi*, *Setaria pallidifusca*, *Setaria plicata*, *Setaria pumila*, *Oplismenus compositus*, *Oplismenus undulatifolius*, *Paspalum dilatatum*, and *Paspalum orbiculare*. By using a combination of morphological traits of the Interdigitating we could distinguishing among the taxa at the genus level for our samples of the studied specimen.

*A. hispidus* (**Supplementary Figure 2**-**II**) produced the Interdigitating with Papillate separated from the main body, Ω-type undulation, articulated connection and rectangular main body. Bilobate could sometimes be found among the Interdigitating. *H. granularis* (**Supplementary Figure 3**-**II**) produced the Interdigitating with Papillate separated from the main body, n-type undulation, articulated connection, and rectangle main body. These two species could be distinguished from other species by the Papillate separated from the main body, this distinct feature allows quick discrimination of *A. hispidus* and *H. granularis*. As these two species belongs to the tribe Andropogoneae, and all other species belong to the tribe Paniceae; this morphological trait might have the potential to be a discriminating feature at the tribe level.

Some of the studied *Digitaria* specimen (n = 3, **Supplementary Figure 7 I**-**III**) produced the Interdigitating with Papillate attached to the main body, smooth type undulation, articulated connection, and rectangle main body. These morphological traits allowed discrimination from other species in our samples. However, in *Digitaria ischaemum* and *Digitaria violascens* (**Supplementary Figure 7**-**IV** and **V**), only the Papillate were silicified to form the disaggregated Papillate, and the other parts of the Interdigitating were very weakly or not silicified. The different phytolith types of *Digitaria* showed the differences within the genus level, which was consistent with the taxonomy: *D. chrysoblephara*, *D. ciliaris*, and *D. sanguinalis* belongs to the section Digitaria, while *D. ischaemum* and *D. violascens* belongs to the section Ischaemum (according to the *Flora Reipublicae Popularis Sinicae*, in Chinese, http://frps.iplant.cn/). The observed differences in our samples showed the potential of a subgenus (section) level discrimination.

All the studied *Setaria* specimen (n = 4, **Supplementary Figure 8**) produced the Interdigitating with Papillate attached to the main body, Ω-type undulation, smooth connection, and rectangle main body, which could be used for discrimination from other species in our samples. The Ω-type undulation observed in the *Setaria* species was mostly from the basic type to Ω-II type; only a few of Ω-III type were observed in the center area of the lemmas in *S. faberi* (**Supplementary Figure 8**-**I**). The Papillate usually grew very large (compared with its main body) in *Setaria* species, thereby affecting the connection part, hampering observation of the connection part, while Papillate in other samples of our studied species did not have such a feature. Some minor morphological traits such as small nodes on the tip of the undulation (**Supplementary Figure 8**-**IV**-**c**) and the flat top of the undulation (**Supplementary Figure 8**-**II**-**b** and **8**-**IV**-**c**) were not used as morphological traits to distinguish samples. As there were limited number of specimens, we could not confirm whether such morphological traits were individual variation or a common feature; however, these minor morphological traits also showed the potential to discriminate among the taxa at species level.

All studied *Oplismenus* specimens (n = 2, **Supplementary Figure 9**-**I** and **II**) produced the Interdigitating with no Papillate, smooth/Ω-type undulation, smooth/articulated connection, and rectangle main body, which could be used for discrimination from other species in our samples. The combination of undulation and connection: smooth undulation with smooth connection, and Ω-type (up to Ω-I type) undulation with articulated connection in the two studied species, suggested the potential of discrimination at the species level.

All studied *Paspalum* specimen (n = 2, **Supplementary Figure 9**-**III** and **IV**) produced the Interdigitating with Papillate attached to the main body, Ω-type undulation, articulated connection, and ovate/rectangle main body. In *Paspalum*, a larger main body than the undulation part was observed to be the identifying feature which was not present in other species. The shape of the main body in the two studied species of this genus, ovate, and rectangle, also suggested the potential of discrimination at the species level.

By using a combination of the morphological traits, we could achieve a reliable discrimination at the genus level among our samples. Although not enough species were studied, the variation in the morphology within the same genus also showed the possibility of discrimination at a more precise level (section or species level). A general distribution pattern of Interdigitating is summarized in **Figure 6**-**A**, which shows that the undulation, connection, and main body all have a continuum variation from small to large along the gradient from the edge to the center.

As described above, *A. hispidus* and *H. granularis* belongs to the tribe Andropogoneae and could be easily discriminated from other species in our samples using the morphological traits of the Interdigitating. In *Setaria* species, parameters w and L could not be measured due to the growth of Papillate. In order to apply the same parameters as in previous studies (Lu et al., 2009b; Ge et al., 2018), morphometric analysis was only applied to *Digitaria*, *Oplismenus*, and *Paspalum* genera. Measurements of the parameters are shown in **Table 4**. The four basic parameters, w, L, h-undulation, and h-body were described as follows: the w value was highest (above 4 μm) in *Paspalum*, while it was low (below 4 μm) in *Digitaria* and *Oplismenus*; the L value was lowest (below 30 μm) in *Digitaria*, higher in *Paspalum* (40–60 μm), and highest in *Oplismenus* (60–90 μm); the h-undulation value showed a large overlap among the three genera and could not be distinguished; the h-body in *Paspalum* had the highest value (10–30 μm), while it was low (below 10 μm) in *Digitaria* and *Oplismenus*. The calculated parameters, h-total, R(w/hu) and R(hu/hb), also varied among the three genera due to the large main body: *Paspalum* had the highest h-total and R(w/hu) values, and the lowest R(hu/hb) value, while in Digitaria and Oplismenus, a large overlap occurred among h-total, R(w/hu), and R(hu/hb). In our samples it could be found that the parameters could vary from species to species, however, parameters were much similar within the same genus and greater differences could be found among different genera, especially when combining all the parameters.

**Figure 7:**
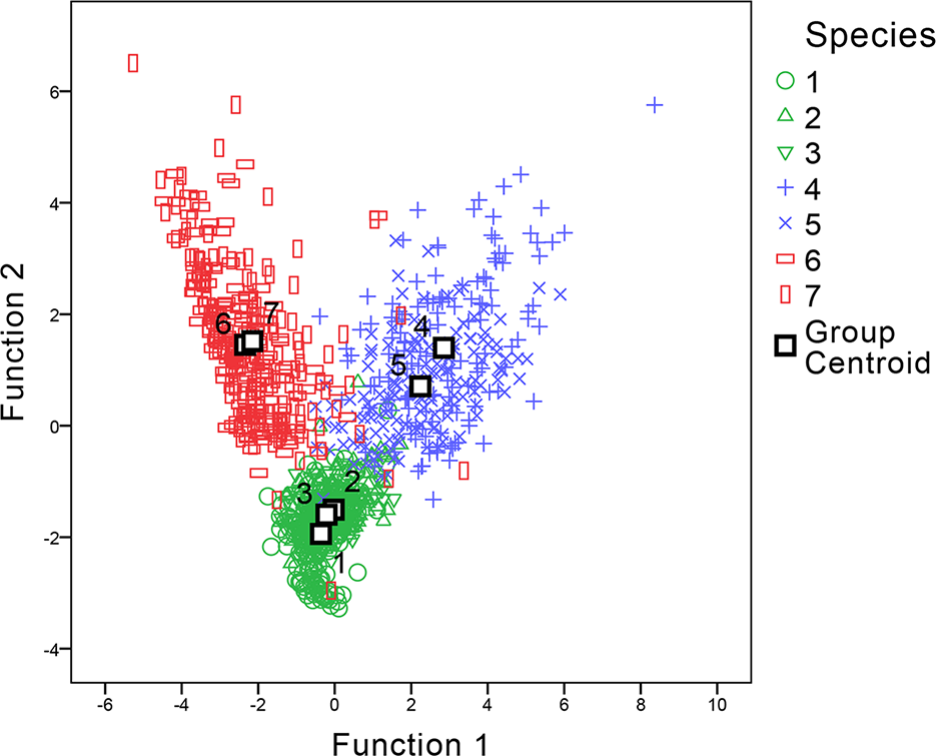
Canonical discriminant analysis of genera *Digitaria*, *Paspalum*, and *Oplismenus*. The number of species refer to 1 *Digitaria sanguinalis*, 2 *Digitaria chrysoblephara*, 3 *Digitaria ciliaris*, 4 *Paspalum orbiculare*, 5 *P. dilatatum*, 6 *Oplismenus compositus*, 7 *Oplismenus undulatifolius*.

For statistical analysis of the parameters, a discriminant analysis was applied to examine if morphometric parameters could aid in distinguishing between the samples. The parameters involved in the discriminant functions included w, L, h-undulation, h-total, R (w/hu), and R (hu/hb) values. The h-body parameter was excluded as it had the largest absolute correlation between each variable and any discriminant functions. Two discriminant functions were generated (shown in **Figure 7**). The discriminant analysis showed that by using these parameters, genus level classification could be achieved among our samples; the genera *Digitaria*, *Oplismenus*, and *Paspalum* could be classified successfully. Furthermore, classification accuracy through cross validation reached 94.3%. However, only 53.9% of the original data could be correctly classified to the species level using the same dataset, suggesting that discrimination at the genus level was much more robust than that at the species level.

## Discussion

### The Interdigitating and the Silica Skeleton

Although it consists of individual phytoliths articulated together, we defined the Interdigitating as a single type of phytolith in this study, as it has a different anatomical origin and morphology than other types of phytoliths. In the previous studies, the term silica skeleton has been used to describe the articulated Elongate dendritic/dentate (Rosen, 1992), which was mostly found in wheat and barley inflorescences, and originates from silicification of epidermal long cells. Previous studies sometimes recognized the Interdigitating and the silica skeleton as the same type of phytolith (Madella et al., 2013; Madella et al., 2014; Weisskopf and Lee, 2016), while we note two major differences: 1. silica skeletons originate from epidermal long cell silicification, while the Interdigitating originate between the epidermal cells and the cuticle layer; 2. silica skeletons are silicified single cells, such as in the Papillate- Elongate dendritic/dentate pattern, and are an assemblage of single phytoliths, while the Interdigitating are an intact layer with interdigitating ornamentation. Thus, we propose that Interdigitating should be defined as a single type of phytolith, a silicon layer with interdigitating ornamentation that covers the surface of a bract. According to this definition, the Bilobate-Elongate dendritic/dentate pattern and the involucre phytoliths layer should belong to the silica skeleton type, as well as other types of phytoliths that originate from silicified epidermal cells.

### Factors Influencing Phytolith Production in Inflorescence Bracts

The Poaceae inflorescence structure is distinct from that of other plants (Berbel et al., 2007), with a more complex organization of bracts under both genetic and molecular control (Kellogg, 2007; Kellogg et al., 2013; Zhang and Yuan, 2014). Thus, the development of phytoliths might also be affected by both genetic and molecular control. Glumes are leaf-like structures that enclose the florets. In the glumes of our samples the phytoliths were generally similar to leaf type phytoliths: Bilobate, Elongate, Acute, and Acute bulbosus (**Table 2**). In the lemmas and paleas of sterile florets, phytolith types tended to be the same as those in glumes; however, in the lemmas and paleas of fertile florets, phytolith types generally differed. The divergence of glumes, lemmas, and paleas is known to be under genetic control (Kellogg, 2007; Kellogg et al., 2013), while the divergence of sterile and fertile florets is mostly under molecular control (Zhang and Yuan, 2014). We found that the types of phytoliths produced in the inflorescence bracts differed among those bracts whose divergence is under genetic control and those under molecular control.

**Figure 8:**
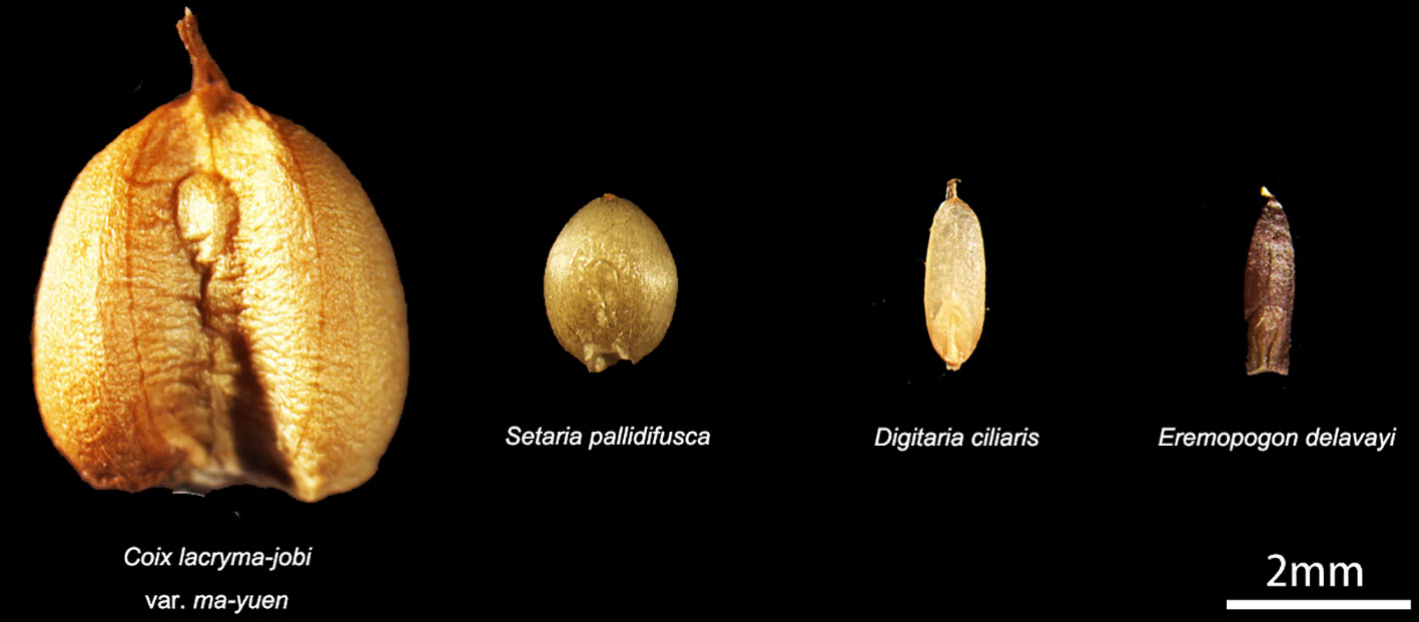
Comparison of seed size. Big and plump seed: *Coix lacryma-jobi* var. *ma-yuen*. Small but plump seed: *Setaria pallidifusca* and *Digitaria ciliaris*. Small and shriveled seed: *Eremopogon delavayi*.

Seed setting requires more energy than flowering and is important for plant regeneration (Bazzaz et al., 2000), thus, seed protection is of great importance to plants. Silicon has been proven to be beneficial to plants (Guntzer et al., 2012) as it aids defense against insects (Massey et al., 2006) and fungi (Remus-Borel et al., 2005); it is presumed that phytoliths in the inflorescence bracts also provide similar effects (Ge et al., 2018). In the present study, we observed that phytoliths were most abundant in bracts from spikelet types II and IV, in which phytoliths cover the surface of the bracts that wrap the seed, and the silicification rate and phytolith quantity are positively correlated with seed size in the studied species. As shown in **Figure 8**, specimens with large, plump seeds, such as Job’s tears (*C. lacryma-jobi* var. *ma-yuen*), produced numerous phytoliths to form the involucre phytolith layer on the involucre surface (spikelet type II); specimens with small, plump seeds, such as *S. pallidifusca*, produced an Interdigitating type covering the lemma and palea (spikelet type IV); specimens with small, shriveled seeds, such as *Eremopogon delavayi*, produced Bilobate and Elongate dendritic/dentate phytoliths on the glume, and a very low number of phytoliths are found on the lemmas and paleas (spikelet type III). As a result, the silica layers (including the involucre phytolith layer and Interdigitating) could provide seed protection, preventing biotic and abiotic harm. Phytolith formation consumes less energy (approximately 1/27) than that required for lignification (Raven, 1983); therefore, species with larger seeds tend to invest additional energy to protect the seeds. As these species require large amounts of energy for seed setting and lignification, phytolith formation could be a relatively economical and effective way to protect seeds.

Lignification also affects phytolith production, as revealed by the hard rind genetic locus (*Hr*) in the genus *Cucurbita* (Piperno et al., 2002). Similar phenomena were observed in the inflorescence bracts of the present study. Strongly lignified bracts, such as the glume of *S. bicolor* and the involucre of *Coix* sp., all produced many more phytoliths than other bracts in the same spikelet. The bracts that produced the Interdigitating were also observed to be of stronger lignification compared to those of other bracts, and very weakly lignified bracts (mostly transparent) did not produce phytoliths at all. As discussed above, a combination of lignification and silicification might be an economical and effective way to provide additional seed protection. Further studies on the genetic control of inflorescence development could facilitate the identification of genes related to phytolith production.

In the current study, more inflorescence phytoliths were observed in species that produced edible seeds (with a larger size and higher seed production rate that would be worth collecting as a food resource), which belonged to the spikelet types II and IV than other species. This corroborated prior observations on other major and minor crops (Ball et al., 2016a). These phenomena suggest the possibility that more inflorescence-type phytoliths might be observed in other unstudied species that possess edible seeds and strongly lignified bracts than those that do not produce edible seeds.

### Discrimination Among Taxa Based on Interdigitating Differences

The Interdigitating was an important phytolith for discriminating among some of the taxa in our samples. Of all the subtypes, the Interdigitating phytolith with n-type undulation was of low taxonomic value; the n-type undulation being the basic type of all undulation patterns (Ge et al., 2018) and was found in both in the inflorescence and leaves of many taxa (Wang and Lu, 1993). Based on the dataset of the present study, the Interdigitating was mostly observed in Panicoideae species (Lu et al., 2009b; Radomski and Neumann, 2011; Zhang et al., 2011; Madella et al., 2013; Kealhofer et al., 2015; Weisskopf and Lee, 2016; Ge et al., 2018).

*A. hispidus* and *H. granularis* from the tribe Andropogoneae produced Interdigitating phytoliths that presented two significant differences from other Interdigitating producers in our samples: *A. hispidus* and *H. granularis* produced the Interdigitating type on the glume and the Papillate type were separated from the main body. Based on the Papillate morphological traits, *A. hispidus* and *H. granularis* could be easily distinguished from other Interdigitating producers, indicating that the Papillate type separated from the main body might be a potential distinguishing morphological trait at the tribe level. Among the rest of the Interdigitating producers in our study, *P. dilatatum* and *P. orbiculare* belong to the tribe Paspaleae. The main body of Interdigitating in these two species are wider than the undulations along the margins, in contrast to other Interdigitating producers where the undulations are larger than the main body. This morphological trait discriminates tribe Paspaleae from tribes Andropogoneae and Paniceae in our samples. Thus, the morphological traits of the Interdigitating: Papillate types separated from the main body and the size of the main body showed great potential for tribe level identification.

Because we only sampled a single specimen of each of the taxa we analyzed in this study, we recognize the need for further analyses of many specimens for each taxon in order to confirm, validate and/or refine our findings. We note that although the number of specimens in the present study was limited, our morphological trait findings were consistent with those reported in other studies. For example, the figures and description provided in a study on *D. ciliaris* (Madella et al., 2013) show that the Interdigitating had Papillate attached to the main body, smooth type undulations, and rectangular main bodies. Another study (Radomski and Neumann, 2011) reported that disaggregated Papillate were abundant in *Digitaria* species (including *D. ciliaris*, *D. exilis*, and *D. iburua*). A study on *Digitaria adscendans* (Weisskopf and Lee, 2016) also reported “short, regular, and cone like papillae.” All of these studies corroborate the present study findings that *Digitaria* species might produce two types of phytoliths in the lemmas and paleas. Further, figures and descriptions of *S. pumila* (Madella et al., 2013; Weisskopf and Lee, 2016), *S. plicata* (Lu et al., 2009b), and *Setaria verticillate* (Madella et al., 2013; Weisskopf and Lee, 2016), show that the Interdigitating all have Papillate attached to the main body, Ω-type undulations (only level-I or II), smooth connections, and rectangular main bodies; all these morphological traits are likewise consistent with the findings of the present study. Similarly, a study on *Paspalum conjugatum* (Weisskopf and Lee, 2016) showed that the Interdigitating had Papillate attached to the main body, Ω-type undulations, articulated connections, and a rectangular main bodies, which again are similar to our findings for *P. dilatatum* in the present study. These prior studies support our findings and confirm the possibility of genus level identification using these morphological traits.

Within the same genus, although some minor morphological commonalities were observed, these morphological traits might not be useful as identification features due to the limited number of studied specimens. The morphometric analysis showed a large overlap within genera (**Table 2** and **Figure 7**), and morphological traits also overlapped among species (Zhang et al., 2011; Kealhofer et al., 2015; Ge et al., 2018; Zhang et al., 2018). However, some minor morphological traits also suggest the possibility of species level identification, but again this must be further evaluated by additional studies.

Morphological traits focusing on the presence of Papillate, undulation pattern, connection shape, and main body shape of Paniceae species are shown in **Table 5** and compared with data from related studies (Lu et al., 2009b; Ge et al., 2018). Thus the use of a combination of morphological traits for genus level discrimination, and species level discrimination is promising.

**Table 5:** Comparison of the Interdigitating phytolith in Tribe Paniceae.

Morphological traits	*Panicum*	*Setaria*			*Echinoloa*	*Digitaria*		*Oplismenus*		*Paspalum*	
	*P. miliaceum*	*S. italica*	*S. viridis*	*other wild Setaria* sp.		*D. sanguinalis, D. chrysoblephara*, *D. ciliaris*	*D. ischaemum, D. violascens*	*O. undulatifolius*	*O. compositus*	*P. orbiculare*	*P. dilatatum*
Papillate	No Papillate	Small or no conical Papillate	Large conical Papillate	Large conical Papillate	No Papillate	Small conical Papillate	Disaggregated Papillate	No Papillate	No Papillate	Small conical Papillate	Small conical Papillate
Undulation	η-type, up to level 3	Ω-type, up to level 3	Ω-type, up to level 3	Ω-type, rarely seen level 3	β-type, up to level 4	Smooth	Non	Ω-type, up to level 1	Smooth	Ω-type, up to level 1	Ω-type, up to level 1
Connection	Articulated, w = 8.95 ± 2.02 R(w/hu) = 0.79 ± 0.12	Articulated, w = 4.37 ± 0.89 R(w/hu) = 0.33 ± 0.11	Smooth, unmeasurable	Smooth, unmeasurable	Articulated, w = 4.86 ± 1.82 R(w/hu) = 0.19 ± 0.09	Articulated, w = 2.53 ± 0.87 R(w/hu) = 0.23 ± 0.13	Non	Articulated, w = 2.58 ± 1.95 R(w/hu) = 0.22 ± 0.17	Smooth, w = 1.50 ± 0.78 R(w/hu) = 0.15 ± 0.12	Articulated, w = 4.82 ± 1.41 R(w/hu) = 0.40 ± 0.20	Articulated, w = 5.58 ± 2.45 R(w/hu) = 0.58 ± 0.31
Main body	Rectangular, R(hu/hb) > 1	Rectangular, R(hu/hb) > 1	Rectangular, R(hu/hb) > 1	Rectangular, R(hu/hb) > 1	Rectangular, R(hu/hb) > 1	Rectangular, R(hu/hb) > 1	Non	Rectangular, R(hu/hb) > 1	Rectangular, R(hu/hb) > 1	Ovate, R(hu/hb) < 1	Rectangular, R(hu/hb) < 1

Data of P. miliaceum and S. italica was adapted from Lu et al. (2009b), data of Echinocloa was adapted from Ge et al. (2018).

Kealhofer et al. (2015) studied the common *Setaria* species in China and doubted the stability of discrimination criteria with regard to discriminating *Setaria* from *Panicum* (Lu et al., 2009b) In their study, they did not apply a combination of morphological traits (apply all traits to the same Interdigitating phytolith), but rather focused on single morphological traits (compare one trait for all Interdigitating phytoliths). The basic n-type and the level-I of Ω-type, η-type, β-type, and smooth type undulation on the edge of the bracts can be highly similar, as highlighted in our previous study (Ge et al., 2018) and this study. As shown in the present study, single morphological traits overlapped at the genus level (**Table 5**), while a combination of morphological traits could provide more robust discrimination at the genus level. In their study (Kealhofer et al., 2015), they also noticed the overlapping occurrence of papillae, the morphology of undulations and connections among different species, thus suggesting that the basic n-type should not be used as a sole identification criterion; their data also revealed the insufficiency of single measurements for differentiating among *Setaria* species, which was consistent with the current study findings. Thus, the key to discrimination is to identify a diagnostic combination of the most common and representative traits, rather than single trait variables. In this study, we identified morphological traits that might be used to discriminate *S. italica* and *S. viridis* from their wild relatives (**Table 5**); however, based our limited sample size, further verification is required. Thus, our findings in this preliminary study support previous published identification criteria for distinguishing between *S. italica* and *P. miliaceum* (Lu et al., 2009b), and the idea that examining a combination of morphological traits has the potential to provide reliable discrimination at the genus level.

### Phytolith Types With High Taxonomic Value in *C. Lacryma-Jobi*

Job’s tears (*C. lacryma-jobi*) is an important plant resource that was used approximately 24,000 years ago (Liu L. et al., 2018). Liu et al. conducted an experiment on starch and phytoliths for their identification in archaeological remains (Liu et al., 2019). In their study, they emphasized cross shaped Bilobate phytoliths found in the glumes, lemmas, paleas, and leaves, which could also be found in other Panicoideae species (their study did not compare other cross shaped Bilobate phytolith producers). In the present study, we found that Rectangular dentate and Blocky amoeboid phytoliths were unique to the inflorescence of this taxon, which has not been reported before. This differentiation has the potential to be the diagnostic criteria for *Coix* identification, even at the subspecies level.

The two specimens of Job’s tears used in this study represented the two commonly used types: edible (with an easy to break involucre) and decorative (with a rigid involucre). The phytoliths differed between the two types (**Supplementary Figure 2**): The edible species (*C. lacryma-jobi* var. *ma-yuen*) produced Bilobate and Elongate phytoliths on the involucre surface which had some height or thickness differences when compared with the flat phytoliths found in the glumes, lemmas, and paleas. The phytolith articulations were not very tight and resulted in an easy to break involucre. However, the decorative species (*C. lacryma-jobi*) produced Blocky amoeboid phytoliths that were tightly connected to form a more rigid phytolith layer on the involucre surface. The different phytolith types on the involucre surface created difficulty in the hulling process; the edible species could be hulled by hand, while the decorative species required the use a hammer. As hulling difficulty would have been an important selection trait (Arora, 1977) in the domestication of Job’s tears, our preliminary results indicated that the Blocky amoeboid involucre phytoliths have a great potential for investigating the domestication process of Job’s tears. As an important minor crop, the domestication of Job’s tears has not been fully studied, partly due to lack of evidence. The different phytolith types on the involucre surface observed in cultivated and wild Job’s tears in this study may provide insight into the domestication process. Again, we note that as these findings were limited to the studied specimens, further studies are needed to validate our findings.

## Conclusions and Perspectives

Phytoliths in every inflorescence bract of 38 common Panicoideae species were observed, various phytolith types were described, and the inflorescence-type of phytoliths were identified. We proposed a new phytolith morphotype, the Interdigitating, and identified several other types of phytoliths, Blocky amoeboid, Rectangular dentate, and Elongate dendritic with multi tent-like arch top, that might be of high taxonomic value. Some of these types we report for the first time in the taxa analyzed in this study. From our observations we suggest that phytoliths in the inflorescence bracts may be positively related to inflorescence development, which might be under both genetic and molecular control. The inflorescence involucre phytolith layer and Interdigitating phytoliths might also be related to a seed protection strategy; species with larger seeds might produce more phytoliths in the outermost bracts to protect the seeds. Thus, species with larger seeds and lignified bracts might have higher potential to produce more phytoliths of higher taxonomic value; more attention should be paid to such species in future studies.

The Interdigitating was an important phytolith type in the inflorescence in our study, especially for millet identification. We summarized Interdigitating morphological traits and found that reliable results for genus level identification among our samples was possible using a combination of morphological traits (**Table 5**). We also found that morphological variation may have the potential for identification at the tribe level (Interdigitating with Papillate separated from the main body and the size of the main body) or species level (morphological variations within the same genus). Again, studies of more specimens are needed for confirmation of the potential. The Blocky amoeboid and Rectangular dentate types from the involucre of Job’s tears have great potential for studying the domestication process of Job’s tears, and the Elongate dendritic with multi tent-like arch top from the glumes of *S. bicolor* might also assist in the identification of sorghum remains. In future studies, more species, more samples per species, and further efforts are needed to provide applicable and robust identification criteria at the tribe/species level.

## Author Contributions

YG and HL designed the research. YG performed the experiment. YG, JZ and CW carried out the image process and data analysis. YG, HL and XG wrote the manuscript. All authors read and approved the final manuscript.

## Funding

This study was jointly supported by the National Natural Science Foundation of China (Grant Nos. 41802021, 41830322 and 41430103), the Strategic Priority Research Program of Chinese Academy of Sciences (Grant No. XDB26000000) and the China Postdoctoral Science Foundation (Grant No. 2018M641480).

## Conflict of Interest

The authors declare that the research was conducted in the absence of any commercial or financial relationships that could be construed as a potential conflict of interest.

## References

[B1] Anderson E. Martin J. H. (1949). World production and consumption of millet and sorghum. Econ. Bot. 3 (3), 265–288. 10.1007/BF02859097

[B2] Arora R. K. (1977). Job’s-tears (coix lacryma-jobi)—a minor food and fodder crop of northeastern India. Econ. Bot. 31 (3), 358–366. 10.1007/bf02866887

[B3] Ball T. B. Gardner J. S. Anderson N. (1999). Identifying inflorescence phytoliths from selected species of wheat (Triticum monococcum, T. dicoccon, T. dicoccoides, and T. aestivum) and barley (Hordeum vulgare and H. spontaneum) (Gramineae). Am. J. Bot. 86 (11), 1615–1623. 10.2307/2656798 10562252

[B4] Ball T. B. Gardner J. S. Anderson N. (2001). An approach to identifying inflorescence phytoliths from selected species of wheat and barley. In Phytoliths: Applications in Earth Sciences and Human History, eds. J. D. Meunier F. Colin (Lisse: Swets & Zeitlinger B.V.), 289–301. 10.1201/NOE9058093455.ch22

[B5] Ball T. B. Ehlers R. Standing M. D. (2009). Review of typologic and morphometric analysis of phytoliths produced by wheat and barley. Breed. Sci. 59 (5), 505–512. 10.1270/jsbbs.59.505

[B6] Ball T. Chandler-Ezell K. Dickau R. Duncan N. Hart T. C. Iriarte J. (2016a). Phytoliths as a tool for investigations of agricultural origins and dispersals around the world. J. Archaeologic. Sci. 68, 32–65.

[B7] Ball T. B. Davis A. Evett R. R. Ladwig J. L. Tromp M. Out W. A. (2016b). Morphometric analysis of phytoliths: recommendations towards standardization from the International Committee for Phytolith Morphometrics. J. Archaeologic. Sci. 68, 106–111. 10.1016/j.jas.2015.03.023

[B8] Ball T. Vrydaghs L. Mercer T. Pearce M. Snyder S. Lisztes-Szabó Z. (2017). A morphometric study of variance in articulated dendritic phytolith wave lobes within selected species of Triticeae and Aveneae. Vegetation History Archaeobot. 26 (1), 85–97. 10.1007/s00334-015-0551-x

[B9] Bazzaz F. A. Ackerly D. D. Reekie E. G. (2000). “Reproductive allocation in plants,” in seeds: The Ecology of Regeneration in Plant Communities, vol. 1–30 Ed. Michael. F. (Oxon & New York: CABI Publishing).

[B10] Bellwood P. (2004). First Farmers: The Origins of Agricultural Societies (Malden (MA): Blackwell).

[B11] Berbel A. Serrano-Mislata A. Benlloch R. Madueño F. (2007). Floral Initiation and Inflorescence Architecture: a comparative view. Ann. Bot. 100 (3), 659–676. 10.1093/aob/mcm146 17679690 PMC2533596

[B12] Blinnikov M. Busacca A. Whitlock C. (2002). Reconstruction of the late Pleistocene grassland of the Columbia basin, Washington, USA, based on phytolith records in loess. Palaeogeogr. Palaeoclimatol. Palaeoecol. 177 (1-2), 77–101. 10.1016/S0031-0182(01)00353-4

[B13] Crawford G. W. (2017). “Plant Domestication in East Asia,” in Handbook of East and Southeast Asian Archaeology. Eds. Habu J. Lape P. V. Olsen. J. W. (New York, NY: Springer New York), 421–435.

[B14] Deng Z. Hung H.C. Carson M. T. Bellwood P. Yang S.l. Lu H. (2018). The first discovery of Neolithic rice remains in eastern Taiwan: phytolith evidence from the Chaolaiqiao site. Archaeologic. Anthropol. Sci. 10 (6), 1477–1484. 10.1007/s12520-017-0471-z

[B15] Denham T. P. Haberle S. G. Lentfer C. Fullagar R. Field J. Therin M. (2003). Origins of agriculture at Kuk Swamp in the Highlands of New Guinea. Science 301 (5630), 189–193. 10.1126/science.1085255 12817084

[B16] Dinda S. Mondal A. K. (2018). The morphometric and numerical analysis of five species of Eragrostis sp. Wolf. based on silica bodies in leaf epidermal cells. Ann. Plant Sci. 7 (5), 2213–2219. 10.21746/aps.2018.7.5.2

[B17] Duncan N. A. Starbuck J. Liu L. (2019). A method to identify Job’s tears, Coix lacryma-jobi L., phytoliths in northern China. J. Archaeologic. Sci.: Rep. 24, 16–23. 10.1016/j.jasrep.2018.12.010

[B18] Dunn R. E. Strömberg C. A. Madden R. H. Kohn M. J. Carlini A. A. (2015). Linked canopy, climate, and faunal change in the Cenozoic of Patagonia. Science 347 (6219), 258–261. 10.1126/science.1260947 25593182

[B19] Ezell K. C. Pearsall D. M. Zeidler J. A. (2006). Root and tuber phytoliths and starch grains document manioc (Manihot esculenta) arrowroot (Maranta arundinacea) and llerén (Calathea sp.) at the real alto site Ecuador. Econ. Bot. 60 (2), 103–120. 10.1663/0013-0001(2006)60[103:RATPAS]2.0.CO;2

[B20] Fuller D. Q. (2006). Agricultural Origins and Frontiers in South Asia: A Working Synthesis. J. World Prehistory 20 (2–4), 127–127. 10.1007/s10963-006-9007-7

[B21] Ge Y. Lu H. Zhang J. Wang C. He K. Huan X. (2018). Phytolith analysis for the identification of barnyard millet (Echinochloa sp.) and its implications. Archaeologic. Anthropol. Sci. 10 (1), 61–73. 10.1007/s12520-016-0341-0

[B22] Gu Y. S. Pearsall D. M. Xie S. C. Yu J. X. (2008). Vegetation and fire history of a Chinese site in southern tropical Xishuangbanna derived from phytolith and charcoal records from Holocene sediments. J. Biogeogr. 35 (2), 325–341. 10.1111/j.1365-2699.2007.01763.x

[B23] Guntzer F. Keller C. Meunier J. D. (2012). Benefits of plant silicon for crops: a review. Agron. Sustain. Dev. 32 (1), 201–213. 10.1007/s13593-011-0039-8

[B24] He K. Lu H. Zheng Y. Zhang J. Xu D. Huan X. (2018). Middle-Holocene sea-level fluctuations interrupted the developing Hemudu culture in the lower Yangtze River, China. Quaternary Sci. Rev. 188, 90–103. 10.1016/j.quascirev.2018.03.034

[B25] Hilbert L. Neves E. G. Pugliese F. Whitney B. S. Shock M. Veasey E. (2017). Evidence for mid-Holocene rice domestication in the Americas. Nat. Ecol. Evol. 1 (11), 1693–1698. 10.1038/s41559-017-0322-4 28993622

[B26] Hodson M. J. Sangster A. G. (1988). Silica deposition in the inflorescence bracts of wheat (Triticum aestivum). I. Scanning electron microscopy and light microscopy. Can. J. Bot. 66 (5), 829–838. 10.1139/b88-121

[B27] Hodson M. J. Parker A. G. Leng M. J. Sloane H. J. (2008). Silicon, oxygen and carbon isotope composition of wheat (Triticum aestivum L.) phytoliths: implications for palaeoecology and archaeology. J. Quaternary Sci. 23 (4), 331–339. 10.1002/jqs.1176

[B28] Horrocks M. Rechtman R. B. (2009). Sweet potato (Ipomoea batatas) and banana (Musa sp.) microfossils in deposits from the Kona Field System, Island of Hawaii. J. Archaeologic. Sci. 36 (5), 1115–1126. 10.1016/j.jas.2008.12.014

[B29] International Committee for Phytolith Taxonomy (ICPT) (2019). (Neumann K. Caroline A. E. Strömberg T. B. Albert R. M. Jenkins E. Vrydaghs Luc. Cummings Linda Scott International Code for Phytolith Nomenclature (ICPN) 2.0; Annals Botany 124 (2), 189–199. 10.1093/aob/mcz064. PMC6758648 31334810

[B30] Jenkins E. (2009). Phytolith taphonomy: a comparison of dry ashing and acid extraction on the breakdown of conjoined phytoliths formed in Triticum durum. J. Archaeologic. Sci. 36 (10), 2402–2407. 10.1016/j.jas.2009.06.028

[B31] Kealhofer L. Huang F. DeVincenzi M. Kim M. M. (2015). Phytoliths in Chinese foxtail millet (Setaria italica). Rev. Palaeobot. Palynol. 223, 116–127. 10.1016/j.revpalbo.2015.09.004

[B32] Kellogg E. Camara P. Rudall P. Ladd P. Malcomber S. Whipple C. (2013). Early inflorescence development in the grasses (Poaceae). Front. Plant Sci. 4 (250), 1–16. 10.3389/fpls.2013.00250 23898335 PMC3721031

[B33] Kellogg E. A. (2007). Floral displays: genetic control of grass inflorescences. Curr. Opin. Plant Biol. 10 (1), 26–31. 10.1016/j.pbi.2006.11.009 17140843

[B34] Liu H. Gu Y. Lun Z. Qin Y. Cheng S. (2018). Phytolith-inferred transfer function for paleohydrological reconstruction of Dajiuhu peatland, central China. Holocene 28 (10), 1623–1630. 10.1177/0959683618782590

[B35] Liu L. Levin M. J. Bonomo M. F. Wang J. Shi J. Chen X. (2018). Harvesting and processing wild cereals in the upper palaeolithic Yellow River valley, China. antiquity 92 (363), 603–619. 10.15184/aqy.2018.36

[B36] Liu L. Duncan N. A. Chen X. Cui J. (2019). Exploitation of job’s tears in Paleolithic and Neolithic China: methodological problems and solutions. Quaternary Int. 529 (20), 25–37. 10.1016/j.quaint.2018.11.019

[B37] Lu H. Y. Liu K. B. (2003). Morphological variations of lobate phytoliths from grasses in China and the south-eastern United States. Diversity Distributions 9 (1), 73–87. 10.1046/j.1472-4642.2003.00166.x

[B38] Lu H. Yang X. Ye M. Liu K. B. Xia Z. Ren X. (2005). Culinary archaeology: millet noodles in Late Neolithic China. Nature 437 (7061), 967–968. 10.1038/437967a 16222289

[B39] Lu H. Y. Wu N. Q. Liu K. B. Jiang H. Liu T. S. (2007). Phytoliths as quantitative indicators for the reconstruction of past environmental conditions in China II: palaeoenvironmental reconstruction in the Loess Plateau. Quaternary Sci. Rev. 26 (5-6), 759–772. 10.1016/j.quascirev.2006.10.006

[B40] Lu H. Zhang J. Liu K. B. Wu N. Li Y. Zhou K. (2009a). Earliest domestication of common millet (Panicum miliaceum) in East Asia extended to 10,000 years ago. Proc. Natl. Acad. Sci. U. S. A. 106 (18), 7367–7372. 10.1073/pnas.0900158106 19383791 PMC2678631

[B41] Lu H. Zhang J. Wu N. Liu K. B. Xu D. Li Q. (2009b). Phytoliths analysis for the discrimination of Foxtail millet (Setaria italica) and Common millet (Panicum miliaceum). PloS One 4 (2), e4448. 10.1371/journal.pone.0004448 19212442 PMC2636886

[B42] Madella M. Lancelotti C. García-Granero J. (2013). Millet microremains—an alternative approach to understand cultivation and use of critical crops in Prehistory. Archaeologic. Anthropol. Sci., 8, 17–28. 10.1007/s12520-013-0130-y

[B43] Madella M. García-Granero J. J. Out W. A. Ryan P. Usai D. (2014). Microbotanical evidence of domestic cereals in Africa 7000 years ago. PloS One 9 (10), e110177. 10.1371/journal.pone.0110177 25338096 PMC4206403

[B44] Massey F. P. Ennos A. R. Hartley S. E. (2006). Silica in grasses as a defence against insect herbivores: contrasting effects on folivores and a phloem feeder. J. Anim. Ecol. 75 (2), 595–603. 10.1111/j.1365-2656.2006.01082.x 16638012

[B45] Novello A. Barboni D. (2015). Grass inflorescence phytoliths of useful species and wild cereals from sub-Saharan Africa. J. Archaeologic. Sci. 59 (0), 10–22. 10.1016/j.jas.2015.03.031

[B46] Parry D. W. Hodson M. (1982). Silica distribution in the caryopsis and inflorescence bracts of foxtail millet [Setaria italica (L.) Beauv.] and its possible significance in carcinogenesis. Ann. Bot. 49 (4), 531–540. 10.1093/oxfordjournals.aob.a086278

[B47] Parry D. W. Smithson F. (1966). Opaline Silica in the inflorescences of some British grasses and cereals. Ann. Bot. 30 (3), 525–538. 10.1093/oxfordjournals.aob.a084094

[B48] Pearsall D. M. Piperno D. R. Dinan E. H. Umlauf R. Zhao Z. J. Benfer R. A. (1995). Distinguishing rice (Oryza-Sativa Poaceae) from wild Oryza species through phytolith analysis—Results of preliminary research. Econ. Bot. 49 (2), 183–196. 10.1007/Bf02862923

[B49] Pearsall D. M. Chandler-Ezell K. Chandler-Ezell A. (2003). Identifying maize in neotropical sediments and soilsusing cob phytoliths. J. Archaeologic. Sci. 30 (5), 611–627. 10.1016/s0305-4403(02)00237-6

[B50] Piperno D. R. Pearsall D. M. (1993). Phytoliths in the reproductive structures of maize and teosinte: implications for the study of maize evolution. J. Archaeologic. Sci. 20 (3), 337–362. 10.1006/jasc.1993.1021

[B51] Piperno D. R. Stothert K. E. (2003). Phytolith evidence for early Holocene Cucurbita domestication in southwest Ecuador. Science 299 (5609), 1054–1057. 10.1126/science.1080365 12586940

[B52] Piperno D. R. Holst I. Wessel-Beaver L. Andres T. C. (2002). Evidence for the control of phytolith formation in Cucurbita fruits by the hard rind (Hr) genetic locus: archaeological and ecological implications. Proc. Natl. Acad. Sci. U. S. A. 99 (16), 10923–10928. 10.1073/pnas.152275499 12149443 PMC125074

[B53] Piperno D. R. Ranere A. J. Holst I. Iriarte J. Dickau R. (2009). Starch grain and phytolith evidence for early ninth millennium B.P. maize from the Central Balsas River Valley, Mexico. Proc. Natl. Acad. Sci. U. S. A. 106 (13), 5019–5024. 10.1073/pnas.0812525106 19307570 PMC2664021

[B54] Piperno D. R. (1984). A comparison and differentiation of phytoliths from maize and wild grasses: use of morphological criteria. Am. Antiquity, 49 (2), 361–383. 10.2307/280024

[B55] Piperno D. R. (1988). Phytolyth Analysis: An Archaeological and Geological Perspective (San Diego: ACADEMIC PRESS, INC). 10.1126/science.241.4873.1694-a 17820900

[B56] Piperno D. R. (2006). Phytoliths: A Comprehensive Guide for Archaeologists and Paleoecologists (Lanham: AltaMira Press).

[B57] Prebble M. Shulmeister J. (2002). An analysis of phytolith assemblages for the quantitativereconstruction of late Quaternary environments of the Lower Taieri Plain,Otago, South Island, New Zealand II. Paleoenvironmentalreconstruction. J. Paleolimnol. 27 (4), 415–427. 10.1023/a:1020314719427

[B58] Prychid C. J. Rudall P. J. Gregory M. (2003). Systematics and biology of silica bodies in monocotyledons. Bot. Rev. 69 (4), 377–440. 10.1663/0006-8101(2004)069[0377:Sabosb]2.0.Co;2

[B59] Radomski K. U. Neumann K. (2011). Grasses and grinding stones: inflorrescence phytoliths from tvioclern West African Poaceae and Archaeological Stone artefacts. Windows Afr. Past: Curr. Approaches Afr. Archaeobot. 3, 153.

[B60] Raven J. A. (1983). The transport and function of silicon in plants. Biol. Rev. Cambridge Philos. Soc. 58 (2), 179–207. 10.1111/j.1469-185X.1983.tb00385.x

[B61] Remus-Borel W. Menzies J. G. Belanger R. R. (2005). Silicon induces antifungal compounds in powdery mildew-infected wheat. Physiol. Mol. Plant Pathol. 66 (3), 108–115. 10.1016/j.pmpp.2005.05.006

[B62] Rosen A. (1992). “Preliminary Identification of Silica Skeletons from Near Eastern Archaeological Sites: An Anatomical Approach,” in Phytolith systematics. Emerging issues. Eds. Rapp G. Jr Mulholland S. C. (New York/London: Plenum), 129–148.

[B63] Rosen A. (1999). “Phytolith analysis in Near Eastern Archaeology,” in The Practical Impact of Science on Aegean and Near Eastern Archaeology. Eds. Pike S. Gitin S. (London: Archetype Press).

[B64] Rosen A. (2004). Phytolith evidence for plant use at Mallaha/Eynan. J. Israel Prehistoric Soc. 34, 189–201.

[B65] Rosen A. Weiner S. (1994). Identifying ancient irrigation: a new method using opaline phytoliths from emmer wheat. J. Archaeologic. Sci. 21 (1), 125–132. 10.1006/jasc.1994.1013

[B66] Rudall P. Prychid C. Gregory T. (2014). Epidermal patterning and silica phytoliths in grasses: an evolutionary history. Bot. Rev. 80 (1), 59–71. 10.1007/s12229-014-9133-3

[B67] Sangster A. G. Hodson M. J. Parry D. W. Rees J. A. (1983). A Developmental-study of silicification in the Trichomes and associated epidermal structures of the inflorescence bracts of the grass, phalaris-canariensis L. Ann. Bot. 52 (2), 171–187. 10.1093/oxfordjournals.aob.a086563

[B68] Schellenberg H. (1908). “Wheat and barley from the North Kurgan, Anau,” in Explorations in Turkestan, vol. 73 . Ed. Pumpelly R. (Washington D.C: Carnegie Institution of Washington), 471–473.

[B69] Stromberg C. A. E. Dunn R. E. Madden R. H. Kohn M. J. Carlini A. A. (2013). Decoupling the spread of grasslands from the evolution of grazer-type herbivores in South America. Nat. Commun. 4 (1478), 1–8. 10.1038/Ncomms2508 23403579

[B70] Stromberg C. A. E. (2005). Decoupled taxonomic radiation and ecological expansion of open-habitat grasses in the Cenozoic of North America. Proc. Natl. Acad. Sci. U. States America 102 (34), 11980–11984. 10.1073/pnas.0505700102 PMC1189350 16099827

[B71] Taxonomy, I.C.f.P (2019). International Code for Phytolith Nomenclature (ICPN) 2.0. Ann. Bot. 10.1093/aob/mcz064 PMC6758648 31334810

[B72] Terrell E. E. Wergin W. P. (1981). Epidermal features and silica deposition in Lemmas and awns of Zizania (Gramineae). Am. J. Bot. 68 (5), 697–707. 10.2307/2442797

[B73] Tubb H. J. Hodson M. J. Hodson G. C. (1993). The inflorescence papillae of the Triticeae—a new tool for taxonomic and archaeological research. Ann. Bot. 72 (6), 537–545. 10.1006/anbo.1993.1142

[B74] Twiss P. C. Suess E. Smith R. M. (1969). Morphological classification of grass phytoliths. Soil Sci. Soc. America Proc. 33 (1), 109. 10.2136/sssaj1969.03615995003300010030x

[B75] Wang Y. J. Lu H. Y. (1993). The study of phytolith and its application (Beijing: China Ocean Press).

[B76] Weisskopf A. Lee G.-A. (2016). Phytolith identification criteria for foxtail and broomcorn millets: a new approach to calculating crop ratios. Archaeologic. Anthropol. Sci. 8 (1), 29–42. 10.1007/s12520-014-0190-7

[B77] Yang X. Barton H. J. Wan Z. Li Q. Ma Z. Li M. (2013). Sago-type palms were an important plant food prior to rice in Southern subtropical China. PloS One 8 (5), e63148. 10.1371/journal.pone.0063148 23667584 PMC3648538

[B78] Yang X. Fuller D. Q. Huan X. Perry L. Li Q. Li Z. (2015). Barnyard grasses were processed with rice around 10000 years ago. Sci. Rep. 5, 16251. 10.1038/srep16251 26536839 PMC4633675

[B79] Yoshida S. Ohnishi Y. Kitagishi K. (1962). Histochemistry of silicon in rice plant: III. The presence of cuticle-silica double layer in the epidermal tissue. Soil Sci. Plant Nutr. 8 (2), 1–5. 10.1080/00380768.1962.10430982

[B80] Zhang D. Yuan Z. (2014). Molecular control of grass inflorescence development. Annu. Rev. Plant Biol. 65 (1), 553–578. 10.1146/annurev-arplant-050213-040104 24471834

[B81] Zhang J. P. Lu H. Y. Wu N. Q. Yang X. Y. Diao X. M. (2011). Phytolith analysis for differentiating between foxtail millet (Setaria italica) and green foxtail (Setaria viridis). PloS One 6 (5), e19726. 10.1371/journal.pone.0019726 21573069 PMC3089634

[B82] Zhang J. Lu H. Liu M. Diao X. Shao K. Wu N. (2018). Phytolith analysis for differentiating between broomcorn millet (Panicum miliaceum) and its weed/feral type (Panicum ruderale). Sci. Rep. 8 (1), 13022. 10.1038/s41598-018-31467-6 30158541 PMC6115419

[B83] Zhao Z. J. Pearsall D. M. Benfer R. A. Piperno D. R. (1998). Distinguishing rice (Oryza sativa poaceae) from wild Oryza species through phytolith analysis, II: Finalized method. Econ. Bot. 52 (2), 134–145. 10.1007/Bf02861201

[B84] Zohary D. Hopf M. Weiss E. (2012). Domestication of Plants in the Old World: The origin and spread of domesticated plants in Southwest Asia, Europe, and the Mediterranean Basin (New York: Oxford University Press).

[B85] Zuo X. Lu H. Li Z. Song B. Xu D. Zou Y. (2016). Phytolith and diatom evidence for rice exploitation and environmental changes during the early mid-Holocene in the Yangtze Delta. Quaternary Res. 86 (3), 304–315. 10.1016/j.yqres.2016.08.001

