# Supplementary material for: Phytoliths in Inflorescence Bracts: Preliminary Results of an Investigation on Common Panicoideae Plants in China
Source: Front Plant Sci. 2020 Feb 20;10:1736. doi: 10.3389/fpls.2019.01736 (PMC7044271; doi:10.3389/fpls.2019.01736)
Supplement: Supplementary file 1 [file DataSheet_1.docx]

**Description of phytoliths types in the inflorescence bracts**

In all the bracts observed, Acute phytoliths (developed from the hair cell walls) were the most commonly observed type of phytoliths, it could be observed almost in all the samples. The amount and silicification of Acute could vary among different species and were almost observed on the center to top areas and the margins of the bracts. Thus, Acute phytoliths were not frequently described in the following texts.

Tribe Andropogoneae

*Apluda mutica* (supplementary figure 1-I)

Phytoliths were observed in the glume and lemma. Phytoliths in the glume including Bilobate concave ends variant1, Polylobate concave ends variant1, Acute and Interdigitating with no Papillate, n-type undulation, articulated ends and rectangular main body, only several Polylobate concave ends variant1 were observed on the center area of the glume. Note the Interdigitating was only weakly silicified. Few of very weakly silicified Acute phytoliths were observed in the lemma, no phytoliths were observed in the palea.

*Arthraxon hispidus* (supplementary figure 1-II)

Phytoliths were observed in the glume and lemma. Phytoliths in the glume including Bilobate convex ends variant saddle-like, Bilobate concave ends variant2, disaggregated Papillate and Interdigitating with no Papillate, Ω-type undulation, articulated connection and rectangular main body. Note the Interdigitating was only weakly silicified and had only Ω-I type undulation. Phytoliths in the lemma were all weakly silicified, only 1 or 2 Bilobate convex ends variant2 and disaggregated Papillate were observed, and the Interdigitating with no Papillate, n-type undulation, articulated connection and rectangular main body were also weakly silicified, and only be observed near the base of the lemma.

*Bothriochloa ischcemum* (supplementary figure 1-III)

Phytoliths were observed in the glume and lemma. Phytoliths in the glume including Bilobate concave ends variant1, Bilobate concave ends variant2 and Interdigitating with no Papillate, n-type undulation, articulated connection and rectangular main body. The Interdigitating was only observed near the base of the glume and was very weakly silicified. The Bilobates were also low in amount and they showed a continuum from variant1 to variant2, and there could be 2 tent-like arch tops of the variant2. In the lemma, only a few of Bilobate convex ends variant1 was observed near the base of the lemma.

*Capillipedium assimile* (supplementary figure 1-IV)

Phytoliths were observed only in the glume. Bilobate concave ends variant1 and Polylobate concave ends variant1 were observed with a low amount. And near the base of the glume, only a few Elongate dendritic/dentate phytoliths were observed, some Bilobate and Polylobate near the base could have convex ends.

*Cymbopogon goeringii* (supplementary figure 1-V)

Phytoliths were observed in the involucre, glume and lemma. Same types of phytoliths were observed in the involucre and glume, while phytoliths in the lemma were different. In the involucre and lemma, Bilobate convex ends variant5/6 and Bilobate convex ends variant saddle-like were observed, sometimes, one end of the Bilobate could be concave. Elongate dendritic/dentate phytoliths were observed near the base of the involucre and glume, and formed a Bilobate- Elongate dendritic/dentate pattern. Near the base of the lemma, Elongate entire and Elongate dendritic/dentate phytoliths were observed with a low amount.

*Eremopogon delavayi* (supplementary figure 1-VI)

Phytoliths were observed in the glume and the awn of lemma. Bilobate convex ends variant1 phytoliths were observed in the glume, sometimes one end of the Bilobate could be concave. Elongate dendritic/dentate and Acute bulbosus phytoliths were observed near the base of the glume, forming a Acute bulbosus-Bilobate- Elongate dendritic/dentate pattern. Only several Acute bulbosus phytoliths were observed at the top of the awn.

*Coix lacryma-jobi* var. *ma-yuen* (supplementary figure 2-I)

This species was the cultivar of Job’s tears and was widely used as food. Phytoliths were abundant and could be found in every bract of the spikelet. In the involucre, Bilobate concave ends variant1, Bilobate concave ends variant5/6, Cross concave ends variant1, Polylobate concave ends variant1, Elongate dendritic/dentate and Rectangular dentate with smooth short sides were observed. All these types were originated from the exterior epidermis except the Rectangular dentate with smooth short sides and a few Bilobate, Cross and Polylobate, which were originated from the inner epidermis. All the phytoliths in the exterior epidermis were tightly connected to form an involucre phytolith layer covering the surface of the involucre. The Elongate dendritic/dentate in the involucre, commonly have the interior surface full of pricks. In the glume, lemma and palea, phytoliths were observed around the center and top parts, where the lignification was stronger, no phytoliths were observed in other parts. In the glume and lemma, Cross concave ends variant1 was the dominant type of phytoliths, Bilobate concave ends variant1 and Polylobate concave ends variant1 could be found in a lower amount. Elongate dendritic/dentate was observed in a low amount in the glume, lemma and palea. Rectangular dentate with protuberant short sides was also observed in a low amount in the glume, lemma and palea, the difference of this type with the Rectangular dentate in the involucre focused on the morphology of the short sides, as in the involucre no Bilobate or Cross phytoliths were produced, the short side thus connected smoothly, while in the glume and lemma, Lobate phytoliths joint with Rectangular dentate together, and resulted in the protuberant short sides. Another difference could be observed on the main body, Rectangular dentate phytoliths in the glume had small granules on the main body, while the Rectangular dentate in the involucre and lemma did not have such granules. In the palea, only Bilobate concave ends variant1 and Cross concave ends variant1 were observed.

*Coix lacryma-jobi* (supplementary figure 2-II)

This species was the wild type of Job’s tears and was mainly used as decoration due to its hard husk (involucre). In the involucre, two types of phytolith were observed, one was the Blocky amoeboid, which was originated from the epidermal cell of the exterior of the involucre; another one was Lobate phytoliths that originated from the interior epidermal cell, including Bilobate concave ends variant1, Cross concave ends variant1 and Polylobate concave ends variant1. The Blocky amoeboid phytoliths were based on a square to rectangular base and a cubic to semi-globular top, with irregular granules on the surface. The Blocky amoeboid phytoliths joint together to form a phytolith layer on the surface of the involucre. In the glume, lemma and palea, Cross concave ends variant1, Bilobate concave ends variant1 and Polylobate concave ends variant1 were the most commonly observed phytoliths types. While only in the glume, a few of Rectangular dentate with granules on surface and protuberant short ends were observed, and only one or two of Elongate dendritic/dentate were observed in the glume.

*Eulalia speciosa* (supplementary figure 3-I)

Same types of phytoliths were observed in the glume and lemma. Bilobate convex ends variant saddle-like and Elongate dendritic/dentate phytoliths formed a Bilobate- Elongate dendritic/dentate pattern in the glume and lemma on the base area.

*Hackelochloa granularis* (supplementary figure 3-II)

Phytoliths were only observed in the glume. In the glume of the fertile floret, Interdigitating with separated Papillate, n-type undulation, articulated connection and rectangular main body was the only type of phytoliths. The main body of this Interdigitating could be bent due to the growth of the Papillate. This Interdigitating covered the surface of the glume, including the pits on the surface of the glume. In the glume of the sterile floret, Acute, Acute bulbosus and Bilobate concave ends variant1 could be observed, the phytoliths types were different from the fertile glume.

*Imperata cylindrica* (supplementary figure 3-III)

Bilobate convex ends variant saddle-like was the only type of phytolith observed in the glume and lemma. Glume produced much more Bilobate than the lemma in amount, and Bilobate in the glume was more strongly silicified than in the lemma.

*Ischaemum anthephoroides* (supplementary figure 3-IV)

Phytoliths were observed in all the bracts. In the glume, Rondel phytoliths were observed near the base of the glume, unlike the Rondel phytoliths commonly observed in subfamily Pooideae, this Rondel phytoliths were very low in amount and have a small chamber in the center. In other areas of the glume, Bilobate convex ends variant saddle-like was observed. In the lemma of the fertile floret, Bilobate convex ends variant1 was observed at the base of the awn (top of the lemma), these Bilobate phytoliths could have very short shank that resulted in a round to ovate outline. On the top of the awn, a few Acute phytoliths could be observed. In the palea of the fertile floret, only a few of Bilobate convex ends variant1 could be observed at the top area of the palea, these Bilobate phytoliths were not fully silicified. In the lemma of the sterile floret, Bilobate convex ends variant1, Acute and Elongate dendritic/dentate phytoliths were observed, the Bilobate and Acute were observed at the top area of the lemma while the Elongate dendritic/dentate was observed at the base area of the lemma. In the palea of the sterile floret, only a few Elongate dendritic/dentate phytoliths were observed at the base area of the palea.

*Microstegium ciliatum* (supplementary figure 4-I)

Phytoliths were observed in the glume and lemma. Bilobate convex ends variant saddle-like was the only type of phytolith in the glume. On the top area of the lemma (near the base of awn), a few Bilobate convex ends variant saddle-like and Elongate dendritic/dentate were observed.

*Microstegium nudum* (supplementary figure 4-II)

Phytoliths were observed in the glume and lemma. Bilobate convex ends variant saddle-like was the main type of phytolith in the glume, near the base of the glume, Elongate dendritic/dentate phytoliths were observed, together with Bilobate phytoliths, they formed the Bilobate- Elongate dendritic/dentate pattern. In the lemma, only a few Acute bulbosus phytoliths were observed on the top of the awn.

*Miscanthus floridulus* (supplementary figure 4-III)

Phytoliths were low in amount in the glume, Bilobate convex ends variant saddle-like and Acute phytoliths were observed at the upper area of the glume. The silicification of the phytoliths was weak. Only a few weakly silicified Bilobate convex ends variant saddle-like were observed at the top area of the lemma.

*Miscanthus nepalensis* (supplementary figure 4-IV)

Phytoliths were observed in the glume and lemma. In the glume, Bilobate convex ends variant saddle-like and Elongate dendritic/dentate phytoliths were the major types of phytoliths. A few of weakly silicified Acute bulbosus phytoliths were observed at the top margin of the glume. In the lemma, a few Bilobate convex variant1 and Elongate dentate cylindric were observed on the central vein at the top area of lemma.

*Miscanthus sinensis* (supplementary figure 4-V)

Same types of phytoliths were observed in the glume and lemma. Bilobate convex ends variant saddle-like and Elongate dendritic/dentate together formed a Bilobate- Elongate dendritic/dentate pattern on the center to top areas of the glume and lemma. On the base area of the glume and lemma, Elongate entire phytoliths were observed in a low amount, and on the top margin area of the glume and lemma, Acute and Acute bulbosus phytoliths were observed.

*Saccharum arundinaceum* (supplementary figure 5-I)

Phytoliths were observed in the glume and lemma. Bilobate convex ends variant saddle-like phytoliths were observed all over the glume, and Elongate dendritic/dentate phytoliths were observed in the bottom area, together they formed the Bilobate- Elongate dendritic/dentate pattern. In the lemma, a few Bilobate convex ends vairant1 and Polylobate convex ends variant1 phytoliths were observed on the top area of the lemma.

*Saccharum rufipilum* (supplementary figure 5-II)

Phytoliths were observed in the glume and lemma. In the glume, Bilobate convex ends variant saddle-like phytoliths were observed all over the glume, and Elongate dendritic/dentate phytoliths were observed on the bottom area of the glume, together they formed the Bilobate- Elongate dendritic/dentate pattern. In the lemma, a few Elongate dendritic/dentate phytoliths were observed on the center vein, and many Acute and Acute bulbosus were observed on the center to top area of the lemma.

*Sorghum bicolor* (supplementary figure 5-III)

This species was widely cultivated and harvested as minor crops in many regions today. Phytoliths were observed in the glume and lemma. In the lower glume, which was thick, Bilobate convex ends variant1, Bilobate convex ends variant5/6, Bilobate convex ends variant saddle-like and Elongate dendritic/dentate with multi-tent like arch top were observed. In the upper glume, which was thin, Bilobate convex ends variant saddle-like and Elongate dendritic/dentate were observed and formed the Bilobate- Elongate dendritic/dentate pattern. Elongate dendritic/dentate phytoliths in the two glumes were different, in the thick lower glume, Elongate dendritic/dentate phytoliths were of 3D structure with multi tent-like arch on top, and the tent-like arch were echinate; in the thin upper glume, Elongate dendritic/dentate phytoliths on the base area had smooth surface, while on the center to top area the surface were full of small granules and were much wider in size. In the lemma, Bilobate convex ends variant saddle-like phytoliths were observed on the base area of the lemma.

*Spodiopogon sibiricus* (supplementary figure 5-IV)

Phytoliths were observed in the glume and lemma. In the glume, Bilobate concave ends variant5/6 and variant saddle-like, as well as Bilobate convex ends variant5/6 and variant saddle-like phytoliths were observed, the Bilobate with concave ends were more in amount. The morphology of the ends of Bilobate could have one convex end and one concave end, and the amount of such transitional morphology was much higher than other Bilobate types. In the lemma, only several Bilobate concave ends variant saddle-like phytoliths were observed on the top area.

*Spodiopogon tainanensis* (supplementary figure 5-V)

Phytoliths were only observed in the glume. Bilobate concave ends variant5/6 and variant saddle-like phytoliths were observed, and only several Elongate dendritic/dentate phytoliths were observed on the base area of the glume.

*Themeda caudata* (supplementary figure 6-I)

Phytoliths were observed in the involucre, glume and lemma. In the involucre and glume, the same types of phytoliths were observed, Bilobate convex ends variant saddle-like and variant7 phytoliths were observed. The variant7 phytoliths were much higher in amount than the variant saddle-like, the variant saddle-like phytoliths were mostly observed on the base and top areas of the glume. In the lemma, only a few Bilobate convex ends variant1 phytoliths were observed on the center to top area.

*Themeda japonica* (supplementary figure 6-II)

Phytoliths were observed in the involucre and glume. Bilobate convex ends variant7, Elongate dendritic/dentate, Elongate entire and weakly silicified Elongate papillar were all observed in the involucre and glume. The weakly silicified Elongate papillar was originated from the epidermal long cells. Prismatic was observed only in the glume, which were originated from the macro hair base on the glume. The amount of Elongate dendritic/dentate was much higher than the Elongate entire cylindric, the Elongate dendritic/dentate phytoliths were mostly observed near the base area, while the Elongate entire cylindric phytoliths were observed on the top area of the involucre and glume.

Tribe Zeugiteae

*Lophatherum gracile* (supplementary figure 6-III)

Phytoliths were observed in the glume, lemma and palea. Bilobate convex ends variant1 and variant saddle-like phytoliths were mostly observed in the glume and lemma, and the amount of vairant1 was higher. On the top of the glume, Acute and Acute bulbosus phytoliths were observed in a low amount, and on the awn of the lemma, several Acute phytoliths were observed. In the palea, only one or two Bilobate convex ends variant1 phytoliths were observed on the center to top area.

Tribe Paniceae

*Digitaria* *chrysoblephara* (supplementary figure 7-I)

Phytoliths were observed in the glume, lemma and palea. In the glume and the lower lemma, Bilobate concave ends variant1 and Elongate dendritic/dentate phytoliths were observed, however, the amount of Elongate dendritic/dentate was low and was only observed on the base area. Sometimes, Bilobate concave ends could have one convex end and one concave end. In the upper lemma and palea, Interdigitating with Papillate attached on the main body, smooth undulation, articulated connecting and rectangular main body was the only type of phytolith observed, and covering the whole surface of the upper lemma and palea.

*Digitaria ciliaris* (supplementary figure 7-II)

Same types of phytoliths were observed as those in the *D. chrysoblephara.*

*Digitaria sanguinalis* (supplementary figure 7-III)

Same types of phytoliths were observed as those in the *D. chrysoblephara.*

*Digitaria ischaemum* (supplementary figure 7-IV)

Phytoliths were observed in the glume, lemma and palea. In the glume and the lower lemma, Bilobate convex ends variant1, Bilobate convex ends variant saddle-like, Polylobate convex ends variant1 and Elongate dendritic/dentate were observed. The Bilobate variant saddle-like and Polylobate phytoliths were much lower in amount compared with Bilobate variant1. The Elongate dendritic/dentate phytoliths were observed on the base area. In the upper lemma and palea, disaggregated Papillate phytoliths were observed. The disaggregated Papillate phytoliths were on the same position as the Papillate attached with main body in the *D. sanguinalis, D. ciliaris and D. chrysoblephara*, the only difference was that the other part (the Interdigitating) was weakly or not silicified, only the Papillate part was silicified.

*Digitaria violascens* (supplementary figure 7-V)

Same types of phytoliths were observed as those in the *Digitaria ischaemum*.

*Setaria faberi* (supplementary figure 8-I)

Phytoliths were observed in the glume, lemma and palea. In the glume and lower lemma, Bilobate concave ends variant1 phytoliths were observed, sometimes, Bilobate with one convex end could be observed in a low amount. In the upper lemma and palea, Interdigitating with Papillate attached with main body, Ω-type undulation, smooth connection and rectangular main body phytoliths were observed covering the surface of the bracts. The Papillate on the Interdigitating grown very large and near to the connection, thus the connection part would be hard to be observed, especially on the center area. The Ω-type undulation was observed mostly to be the Ω-I and Ω-II type, only in the center of the upper lemma, a few Ω-III type undulation phytoliths were observed.

*Setaria pallidifusca* (supplementary figure 8-II)

Phytoliths were observed in the glume, lemma and palea. In the glume and lower lemma, Bilobate convex ends variant1 phytoliths were observed. In the upper lemma and palea, Interdigitating with Papillate attached with main body, Ω-type undulation, smooth connection and rectangular main body phytoliths were observed covering the surface of the bracts. The Papillate on the Interdigitating grown very large and near to the connection, thus the connection part would be hard to observe, especially on the center area. Un like the Ω-type undulation in *S. faberi*, no Ω-II type undulation phytoliths were observed, and the top of the undulation was flat.

*Setaria plicata* (supplementary figure 8-III)

Phytoliths were observed in the glume, lemma and palea. In the glume and lower lemma, Bilobate concave ends variant1 phytoliths were observed, sometimes, Bilobate with one convex end could be observed in a low amount. In the upper lemma and palea, Interdigitating with Papillate attached with main body, Ω-type undulation, smooth connection and rectangular main body phytoliths were observed covering the surface of the bracts. The Papillate on the Interdigitating grown very large and near to the connection, thus the connection part would be hard to observe, especially on the center area. The Ω-type undulation was observed to be the Ω-I type, no Ω-II type undulation phytoliths were observed.

*Setaria pumila* (supplementary figure 8-IV)

Phytoliths were observed in the glume, lemma and palea. In the glume and lower lemma, Bilobate convex ends variant1 phytoliths were observed. In the upper lemma and palea, Interdigitating with Papillate attached with main body, Ω-type undulation, smooth connection and rectangular main body phytoliths were observed covering the surface of the bracts. The Papillate on the Interdigitating grown very large and near to the connection, thus the connection part would be hard to observe, especially on the center area. Un like the Ω-type undulation in *S. faberi*, no Ω-II type undulation phytoliths were observed, and the top of the undulation was flat on the center of the upper lemma, notably, small nodes could be observed on the top of the undulation on the center parts of the upper lemma and palea.

*Oplismenus undulatifolius* (supplementary figure 9-I)

Phytoliths were observed in the glume, lemma and palea. In the glume, Bilobate concave ends variant1 was the most common Bilobate phytolith, other Cross and Polylobate and other variants were all observed in a low amount. Elongate dendritic/dentate phytoliths were also observed in a low amount on the base area of the glume. Similar to the Elongate, Interdigitating with no Papillate, n-type undulation, smooth connection and rectangular main body was observed on the base to center area in the glume. In the lower lemma and palea (bracts of the sterile floret), Cross concave ends variant1 was the most common Lobate phytoliths, the Polylobate phytoliths were one side three lobes and one side two lobes and resulted in the shape of a star. Elongate dendritic/dentate and Interdigitating with no Papillate, n-type undulation, smooth connection and rectangular main body were observed on the base to center area in the lower lemma and palea in a low amount. In the upper lemma, Interdigitating with no Papillate, Ω-type undulation, articulated connection and rectangular main body was the only type of phytoliths. The morphology of undulation was the basic Ω-type, only a few of Ω-I undulation were observed even on the center area of the upper lemma. In the upper palea, Interdigitating with no Papillate, Ω-type undulation, smooth connection and rectangular main body was the only type of phytolith. The morphology of undulation was the basic Ω-type, even on the center area of the upper palea. It could be found that the presence of Ω-I type on the center area of upper lemma and the articulated connection were the major differences of the Interdigitating between the upper lemma and upper palea.

*Oplismenus compositus* (supplementary figure 9-I)

Phytoliths were observed in the glume, lemma and palea. In the glume and lower lemma, Bilobate concave ends variant1, Cross concave ends variant1 and Polylobate concave ends variant1 were observed, of which Cross concave ends variant1 was the most common type of lobate phytoliths. In the upper lemma and palea, Interdigitating with no Papillate, smooth undulation, smooth connection, rectangular main body was the only type of phytoliths that covering the whole bracts.

Tribe Paspaleae

*Paspalum* *orbiculare* (supplementary figure 9-I)

Phytoliths were observed in the glume, lemma and palea. In the glume and the lower lemma, Polylobate concave ends variant1 was the most common phytoliths type observed. This Polylobate had one side three lobes and the other side two lobes, and formed a shape of a star, and the Bilobate concave ends variant1 was only observed in a very low amount. In the upper lemma and palea, Interdigitating with Papillate attached to the main body, Ω-type undulation, articulated connection and ovate main body was the main type of phytolith. Although the undulation was the Ω-type, it could be found that only the basic Ω-type was observed, even on the center area. The ovate main body was so far the first observed non-rectangular main body of all Interdigitating. On the very top of the upper lemma and palea, only a few Bilobate concave ends variant2, Cross concave ends variant2 and Polylobate convex ends variant2 phytoliths were observed.

*Paspalum dilatatum* (supplementary figure 9-I)

Phytoliths were observed in the glume, lemma and palea, and the types of phytoliths were almost the same with those in the *P. orbiculare*. The only difference was the shape of the main body of Interdigitating on the upper lemma and palea. The Interdigitating in *P. dilatatum* had a rectangular main body, compare with the ovate main body of the *P. orbiculare*.

**Supplementary Figure 1** Illustration of phytolith morphology in the inflorescence of *Apluda mutica, Arthraxon hispidus, Bothriochloa ischcemum, Capillipedium assimile, Cymbopogon goeringii,* and *Eremopogon delavayi*.

**I** *Apluda mutica*, **a** Bilobate concave ends variant1, Acute, Interdigitating with no Papillate, n-type undulation, articulated connection and rectangular main body phytoliths in the glume; **b** Bilobate concave ends variant1 in the glume, side view; **c** Polylobate concave ends variant1 and one Polylobate phytolith in the lemma.

**II** *Arthraxon hispidus*, **a** Bilobate concave ends variant2, disaggregated Papillate and Interdigitating with no Papillate, Ω-I type undulation, articulated connection and rectangular main body phytoliths in the glume; **b** Bilobate convex ends variant2, disaggregated Papillate and Interdigitating with no Papillate, Ω-type undulation, articulated connection and rectangular main body in the glume; **c** Interdigitating with no Papillate, n-type undulation, articulated connection and rectangular main body in the lemma.

**III** *Bothriochloa ischcemum*, **a** Bilobate concave ends variant1 and Interdigitating with no Papillate, n-type undulation, articulated connection and rectangular main body in the glume; **b** Bilobate concave ends variant2 in the glume; **c** Bilobate convex ends variant1 in the lemma.

**IV** *Capillipedium assimile*, **a** Bilobate concave ends variant1 and Polylobate concave ends variant1 on the base area of the glume; **b** weakly silicified Bilobate concave ends variant1 and Polylobate concave ends variant1 on the center area of the glume; **c** Elongate dendritic/dentate on the base area of the glume.

**V** *Cymbopogon goeringii*, **a** Bilobate convex ends variant5/6 and Bilobate convex ends variant saddle-like phytoliths in the involucre and glume; **b** the Bilobate- Elongate dendritic/dentate pattern in the involucre and glume; **c** Elongate entire and Elongate dendritic/dentate on the base area of the lemma.

**VI** *Eremopogon delavayi*, **a** Bilobate convex ends variant1 phytoliths in the glume; **b** Acute bulbosus-Bilobate- Elongate dendritic/dentate pattern in the glume; **c** Acute bulbosus phytoliths on the top of the awn.

**Supplementary Figure 2** Illustration of phytolith morphology in the inflorescence of *Coix lacryma-jobi var. ma-yuen* and *Coix lacryma-jobi*.

**I** *Coix lacryma-jobi var. ma-yuen*, **a** the surface silicified cell layer on the involucre, comprised of Bilobate concave ends variant1, Bilobate concave ends variant5/6, Elongate dendritic/dentate top view; **b** Elongate dendritic/dentate bottom view; **c** Rectangular dentate with smooth short sides in the involucre; **d** Cross concave ends variant1, Bilobate concave ends variant1 and Polylobate concave ends variant1 phytoliths in the glume; **e** Rectangular dentate with protuberant short sides in the glume; **f** Cross concave ends variant1, Bilobate concave ends variant1, Polylobate concave ends variant1 and Elongate dendritic/dentate in the lemma; **g** Cross concave ends variant1, Bilobate concave ends variant1, Polylobate concave ends variant1 and Rectangular dentate with protuberant short sides in the lemma; **h** and **i** Cross concave ends variant1, Bilobate concave ends variant1 and Polylobate concave ends variant1 in the palea.

**II** *Coix lacryma-jobi*, **a** the silicified cell layer on the surface of the involucre, top view; **b** bottom view of the Blocky amoeboid; **c** Cross concave ends variant1, Bilobate concave ends variant1 and Polylobate concave ends variant1 in the involucre; **d** Cross concave ends variant1, Bilobate concave ends variant1 and Rectangular dentate in the glume; **e** Cross concave ends variant1 and Elongate dendritic/dentate in the glume **f** side view of the Polylobate concave ends variant1 in the glume; **g** Cross concave ends variant1, Bilobate concave ends variant1 and Polylobate concave ends variant1 in the lemma; **h** Cross concave ends variant1 and the one side convex one side concave lobate phytoliths in the lemma; **i** Cross concave ends variant1 and Bilobate concave ends variant1 in the palea.

**Supplementary Figure 3** Illustration of phytolith morphology in the inflorescence of *Eulalia speciosa*, *Hackelochloa granularis*, *Imperata cylindrica*, and *Ischaemum anthephoroides*.

**I** *Eulalia speciosa*, **a** the Bilobate- Elongate dendritic/dentate pattern in the glume; **b** side view of the Bilobate convex ends variant saddle-like in the glume, **c** the Bilobate- Elongate dendritic/dentate pattern in the lemma.

**II** *Hackelochloa granularis*, **a** the Interdigitating on the surface of the glume of the fertile floret, the center of the figure showed the pit structure on the glume; **b** the Interdigitating with separated Papillate, n-type undulation, articulated connection and rectangular main body on the glume of the fertile floret; **c** Acute, Acute bulbosus and Bilobate concave ends variant1 on the glume of sterile floret.

**III** *Imperata cylindrica*, **a** Bilobate convex ends variant saddle-like in the glume; **b** Bilobate convex ends variant saddle-like, side view, **c** Bilobate convex ends variant saddle-like in the lemma.

**IV** *Ischaemum anthephoroides*, **a** Rondel phytoliths with a small chamber in the center on the base of the glume; **b** Bilobate convex ends variant saddle-like on the glume; **c** Bilobate convex ends variant saddle-like, side view; **d** Bilobate convex ends variant1 and Acute phytoliths on the top area of the lemma of sterile floret; **e** Elongate dendritic/dentate phytolith on the base to center area of the lemma of the sterile floret; **f** Elongate dendritic/dentate on the palea of the sterile floret; **g** Bilobate convex ends variant1 on the base of the awn on the lemma of the fertile floret; **h** very weakly silicified Acute on the top of the awn on the lemma of the fertile floret; **i** Bilobate convex ends variant1 on the palea of the fertile floret.

**Supplementary Figure 4** Illustration of phytolith morphology in the inflorescence of *Microstegium ciliatum, Microstegium. nudum, Miscanthus floridulus, Miscanthus nepalensis,* and *Miscanthus sinensis*.

**I** *Microstegium ciliatum*, **a** Bilobate convex ends variant saddle-like in the glume; **b** Bilobate convex ends variant saddle-like, side view; **c** Bilobate convex ends variant saddle-like and Elongate dendritic/dentate on the top area of the lemma.

**II** *Microstegium nudum*, **a** the Bilobate- Elongate dendritic/dentate pattern on the glume; **b** Bilobate convex ends variant saddle-like, side view; **c** Acute bulbosus on the awn of the lemma.

**III** *Miscanthus floridulus*, **a** Bilobate convex ends variant saddle-like in the glume; **b** Bilobate convex ends variant saddle-like, side view; **c** Acute on the top area of the glume.

**IV** *Miscanthus nepalensis*, **a** the Bilobate- Elongate dendritic/dentate pattern on the glume; **b** Acute bulbosus on the glume, **c** Bilobate convex ends variant1, Polylobate convex ends variant1 and Elongate dentate cylindric on the lemma.

**V** *Miscanthus sinensis*, **a** the Bilobate- Elongate dendritic/dentate pattern on the glume; **b** Elongate entire on the glume; **c** Bilobate convex ends variant saddle-like, Acute and Acute bulbosus on the top area of the glume; **d** the Bilobate- Elongate dendritic/dentate pattern on the lemma; **e** Elongate entire on the lemma; **f** Bilobate convex ends variant saddle-like, Acute and Acute bulbosus on the top area of the lemma.

**Supplementary Figure 5** Illustration of phytolith morphology in the inflorescence of *Saccharum arundinaceum, Saccharum rufipilum,* *Sorghum bicolor,* *Spodiopogon sibiricus* and *Spodiopogon tainanensis*.

**I** *Saccharum arundinaceum*, **a** the Bilobate- Elongate dendritic/dentate pattern on the glume; **b**, Bilobate convex ends variant saddle-like, side view; **c** Bilobate convex ends variant1 and Bilobate convex ends variant1 on the lemma.

**II** *Saccharum rufipilum*, **a** the Bilobate- Elongate dendritic/dentate pattern on the glume; **b** Elongate dendritic/dentate, Acute and Acute bulbosus phytoliths on the lemma; **c** Acute bulbosus phytoliths on the top of the lemma.

**III** *Sorghum bicolor*, **a** Bilobate convex ends variant1, Bilobate convex ends variant saddle-like and Elongate dendritic/dentate phytoliths on the lower glume; **b** Bilobate- Elongate dendritic/dentate pattern on the upper glume; **c** Elongate dendritic/dentate multi tent-like arch phytoliths on the lower glume, top view; **d** Elongate dendritic/dentate multi tent-like arch phytoliths in the lower glume, side view, **e** Bilobate convex ends variant 5/6 on the lower glume; **f** Bilobate convex ends variant saddle-like on the lemma.

**IV** *Spodiopogon sibiricus*, **a** Bilobate concave ends variant5/6 on the glume; **b** Bilobate concave ends variant saddle-like on the glume, side view; **c** Bilobate concave ends variant saddle-like on the lemma.

**V** *Spodiopogon tainanensis*, **a** Bilobate concave ends variant5/6 on the glume; **b** Elongate dendritic/dentate on the glume; **c** Bilobate concave ends variant saddle-like on the glume, side view.

**Supplementary Figure 6** Illustration of phytolith morphology in the inflorescence of *Themeda caudata, Themeda japonica* and *Lophatherum gracile*.

**I** *Themeda caudata*, **a** Bilobate convex ends variant saddle-like and Bilobate convex ends variant7 on the glume; **b** Bilobate convex ends variant7 (top left) and Bilobate convex ends variant saddle-like (lower right), amplified view; **c** Bilobate convex ends variant1 on the lemma.

**II** *Themeda japonica*, **a** Bilobate convex ends variant7 on the glume; **b** Bilobate convex ends variant7, amplified view; **c** Bilobate convex ends variant7 and some weakly silicified Elongate papillar on the glume; **d** Prismatic silicified hair base on the glume; **e** Elongate dendritic/dentate on the bottom area of the glume, **f** Elongate entire cylindric on the top area of the glume.

**III** *Lophatherum gracile*, **a** Acute and Acute bulbosus on the top margin of the glume; **b** Bilobate convex ends variant1 and variant saddle-like on the glume; **c** Bilobate convex ends variant saddle-like, side view; **d** Acute on the awn of the lemma; **e** Bilobate convex ends variant1 and variant saddle-like on the lemma, **f** Bilobate convex ends variant1 on the palea.

**Supplementary Figure 7** Illustration of phytolith morphology in the inflorescence of *Digitaria chrysoblephara, Digitaria ciliaris, Digitaria sanguinalis, Digitaria ischaemum,* and *Digitaria violascens*.

**I** *Digitaria chrysoblephara*, **a** Bilobate concave ends variant1 on the glume; **b** Interdigitating with Papillate attached to the main body, smooth undulation, articulated connection and rectangular main body on the upper palea; **c** Interdigitating with Papillate attached to the main body, smooth undulation, articulated connection and rectangular main body on the upper lemma.

**II** *Digitaria ciliaris*, **a** Bilobate concave ends variant1 and Elongate dendritic/dentate on the glume; **b** Interdigitating with Papillate attached to the main body, smooth undulation, articulated connection and rectangular main body on the upper palea; **c** Interdigitating with Papillate attached to the main body, smooth undulation, articulated connection and rectangular main body on the upper lemma.

**III** *Digitaria sanguinalis*, **a** Bilobate concave ends variant1 on the glume; **b** Interdigitating with Papillate attached to the main body, smooth undulation, articulated connection and rectangular main body on the upper palea; **c** Interdigitating with Papillate attached to the main body, smooth undulation, articulated connection and rectangular main body on the upper lemma.

**IV** *Digitaria ischaemum*, **a** Bilobate convex ends variant1 and Polylobate convex ends variant1 on the glume; **b** disaggregated Papillate on the upper palea; **c** disaggregated Papillate on the upper lemma.

**V** *Digitaria violascens*, **a** Bilobate convex ends variant1, Bilobate convex ends variant saddle-like and Polylobate concave ends variant1 on the glume upper palea; **b** disaggregated Papillate on the; **c** disaggregated Papillate on the upper lemma.

**Supplementary Figure 8** Illustration of phytolith morphology in the inflorescence of *Setaria. faberi*, *Setaria pallidifusca*, *Setaria plicata* and *Setaria pumila*.

**I** *Setaria faberi*, **a** Bilobate concave ends variant1 on the glume; **b** Interdigitating with Papillate attached with main body, Ω-type undulation, smooth connection and rectangular main body on the base area of the upper lemma; **c** Interdigitating with Papillate attached with main body, Ω-type undulation, smooth connection and rectangular main body on the top area of the upper lemma; **d** Interdigitating with Papillate attached with main body, Ω-type undulation, smooth connection and rectangular main body on the center area of the upper lemma; **e** **and f** Interdigitating with Papillate attached with main body, Ω-type undulation, smooth connection and rectangular main body on the center area of the upper palea.

**II** *Setaria pallidifusca*, **a** Bilobate convex ends variant1 on the glume; **b** Interdigitating with Papillate attached with main body, Ω-type undulation, smooth connection and rectangular main body on the base to center area of the upper lemma; **c** Interdigitating with Papillate attached with main body, Ω-type undulation, smooth connection and rectangular main body on the center area of the upper lemma, showing a flat top of the undulation.

**III** *Setaria plicata*, **a** Bilobate concave ends variant1 on the glume; **b** Interdigitating with Papillate attached with main body, Ω-type undulation, smooth connection and rectangular main body on the center area of the upper palea; **c** Interdigitating with Papillate attached with main body, Ω-type undulation, smooth connection and rectangular main body on the center area of the upper lemma.

**IV** *Setaria pumila*, **a** Bilobate convex ends variant1 on the glume; **b** Interdigitating with Papillate attached with main body, Ω-type undulation, smooth connection and rectangular main body on the center area of the upper palea; **c** Interdigitating with Papillate attached with main body, Ω-type undulation, smooth connection and rectangular main body on the center area of the upper lemma, showing the small nodes on the flat top of the undulation.

**Supplementary Figure 9** Illustration of phytolith morphology in the inflorescence of *Oplismenus undulatifolius,* *Oplismenus compositus, Paspalum orbiculare* and *Paspalum dilatatum*.

**I** *Oplismenus undulatifolius*, **a** Bilobate concave ends variant1 (top most), Cross concave ends variant1 (lower most), Cross concave ends variant5/6 (right most) and Polylobate concave ends variant1 (center and left most) on the glume; **b** Cross concave ends variant1 and Interdigitating with no Papillate, n-type undulation, smooth connected and rectangular main body on the base area of the glume; **c** Cross concave ends variant1, Polylobate concave ends variant1 and Elongate dendritic/dentate on the lemma and palea of sterile floret; **d** Cross concave ends variant1 and Interdigitating with no Papillate, n-type undulation, smooth connected and rectangular main body on the base area of the lemma and palea of the sterile floret; **e** Interdigitating with no Papillate, Ω-type undulation, articulated connection and rectangular main body on the lemma of the fertile floret; **f** Interdigitating with no Papillate, Ω-type undulation, articulated connection and rectangular main body on the palea of the fertile floret.

**II** *Oplismenus undulatifolius*, **a** Cross concave ends variant1 and Polylobate concave ends variant1; **b** Interdigitating with no Papillate, smooth undulation, smooth connection and rectangular main body on the lemma; **c** Interdigitating with no Papillate, smooth undulation, smooth connection and rectangular main body on the lemma.

**III** *Paspalum orbiculare*, **a** Polylobate concave ends variant1 and Bilobate concave ends variant1 (next to right most) on the glume; **b** Polylobate concave ends variant2 and Bilobate concave ends variant2 on the top area of the lemma; **c** Interdigitating with Papillate attached to the main body, Ω-type undulation, articulated connection and ovate main body on the base area of the lemma; **d** Interdigitating with Papillate attached to the main body, Ω-type undulation, articulated connection and ovate main body on the center area of the lemma; **e** Interdigitating with Papillate attached to the main body, Ω-type undulation, articulated connection and ovate main body on the center area of the palea; **f** Interdigitating with Papillate attached to the main body, Ω-type undulation, articulated connection and ovate main body on the top area of the palea.

**IV** *Paspalum dilatatum*, **a** Bilobate concave ends variant1 (right middle) and Polylobate concave ends variant1 on the glume; **b** Interdigitating with Papillate attached to the main body, Ω-type undulation, articulated connection and rectangular main body on the base area of the lemma; **c** Interdigitating with Papillate attached to the main body, Ω-type undulation, articulated connection and rectangular main body on the center area of the lemma.
