# Supplementary figures and images for: Phytoliths in Inflorescence Bracts: Preliminary Results of an Investigation on Common Panicoideae Plants in China
Source: Front Plant Sci. 2020 Feb 20;10:1736. doi: 10.3389/fpls.2019.01736 (PMC7044271; doi:10.3389/fpls.2019.01736)

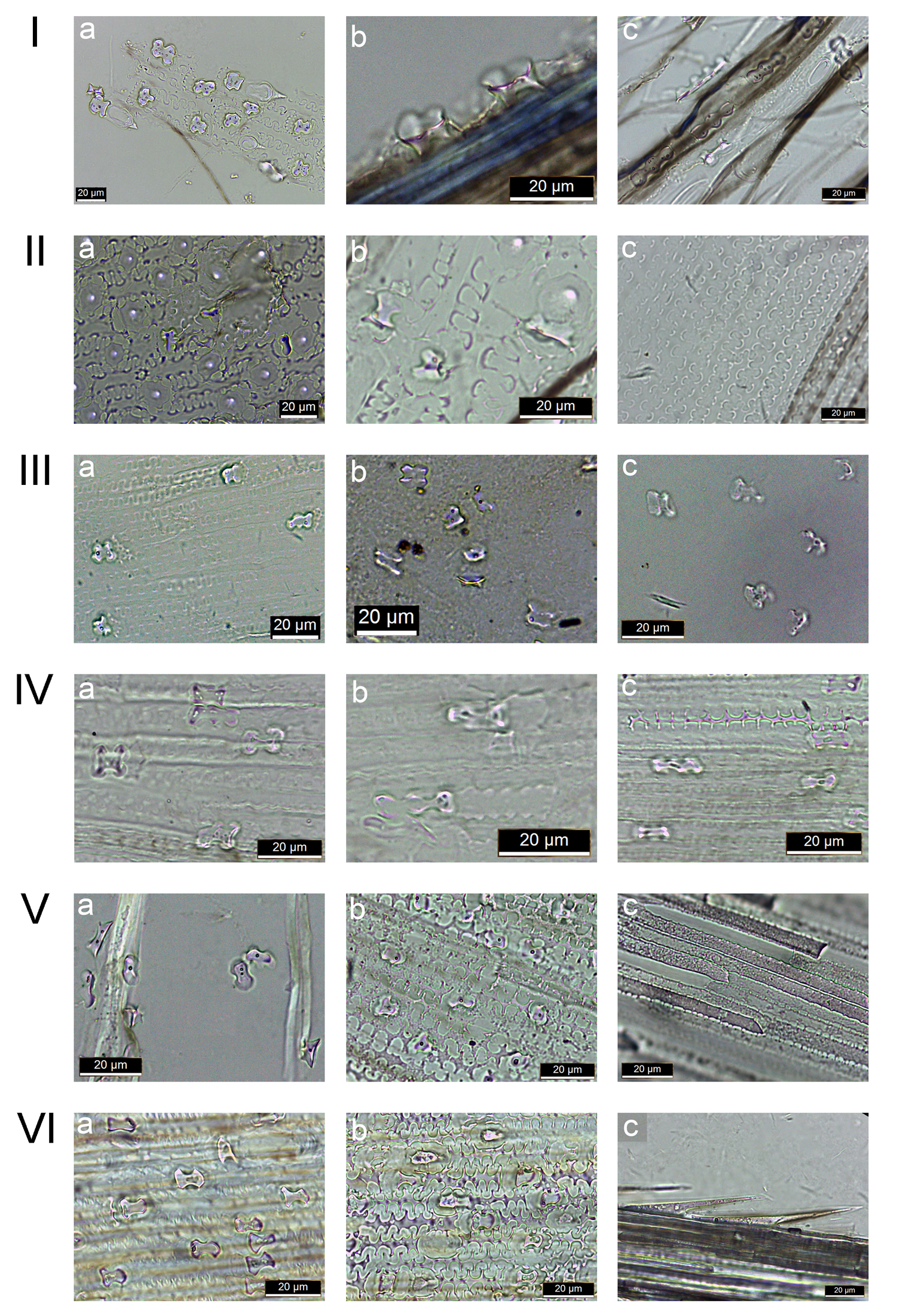

Supplement: Supplementary file 2 [file Image_1.jpeg]

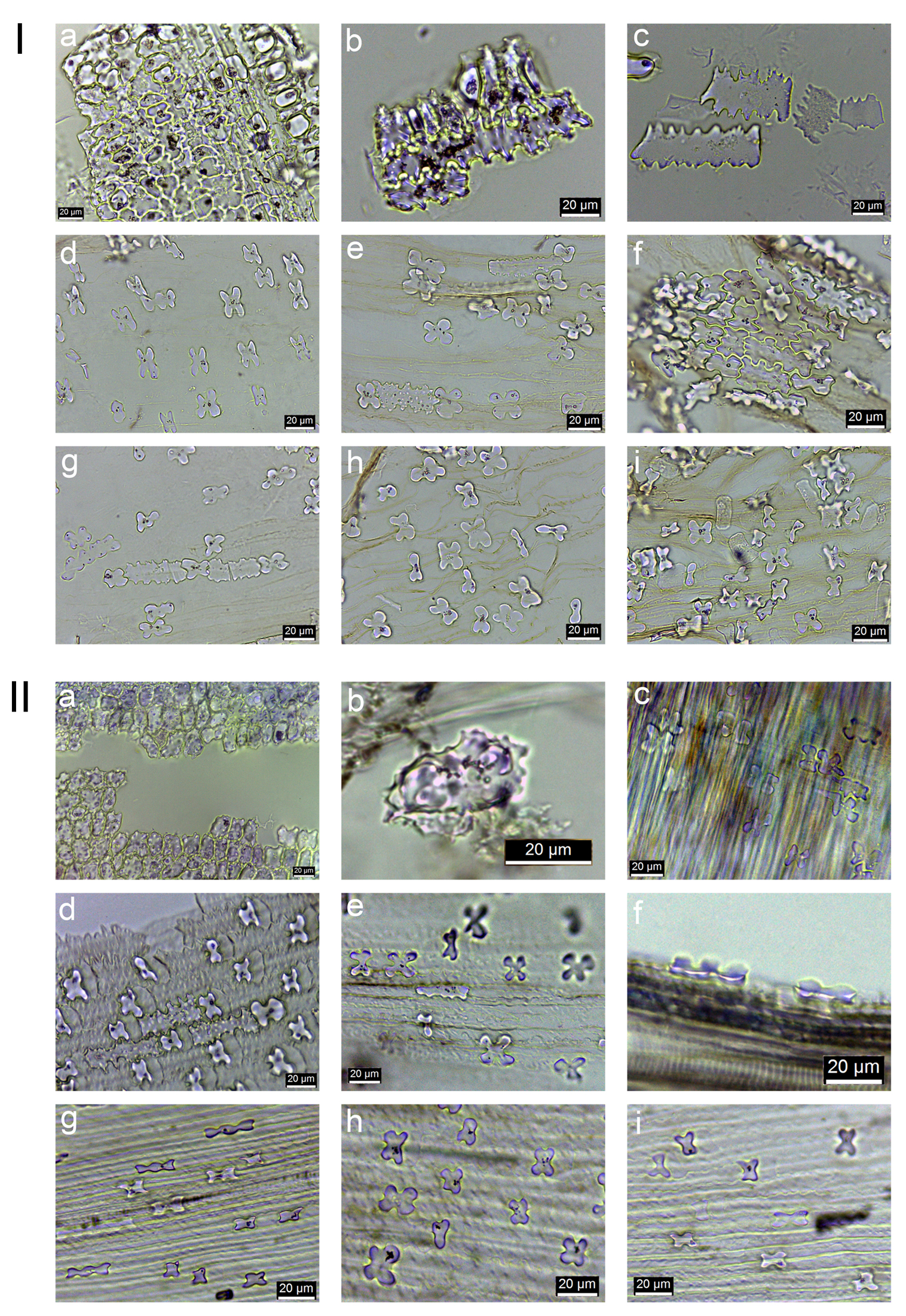

Supplement: Supplementary file 3 [file Image_2.jpeg]

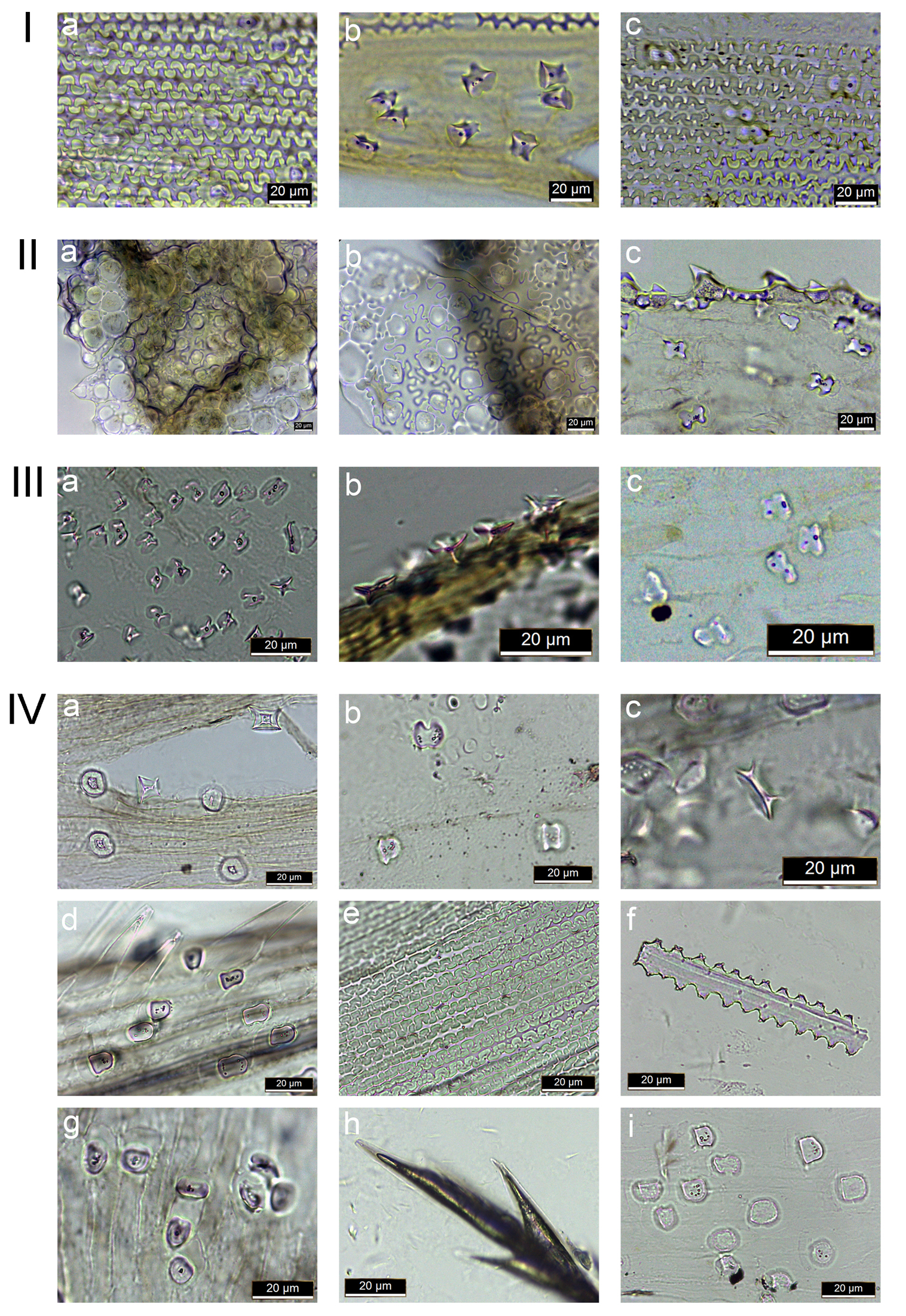

Supplement: Supplementary file 4 [file Image_3.jpeg]

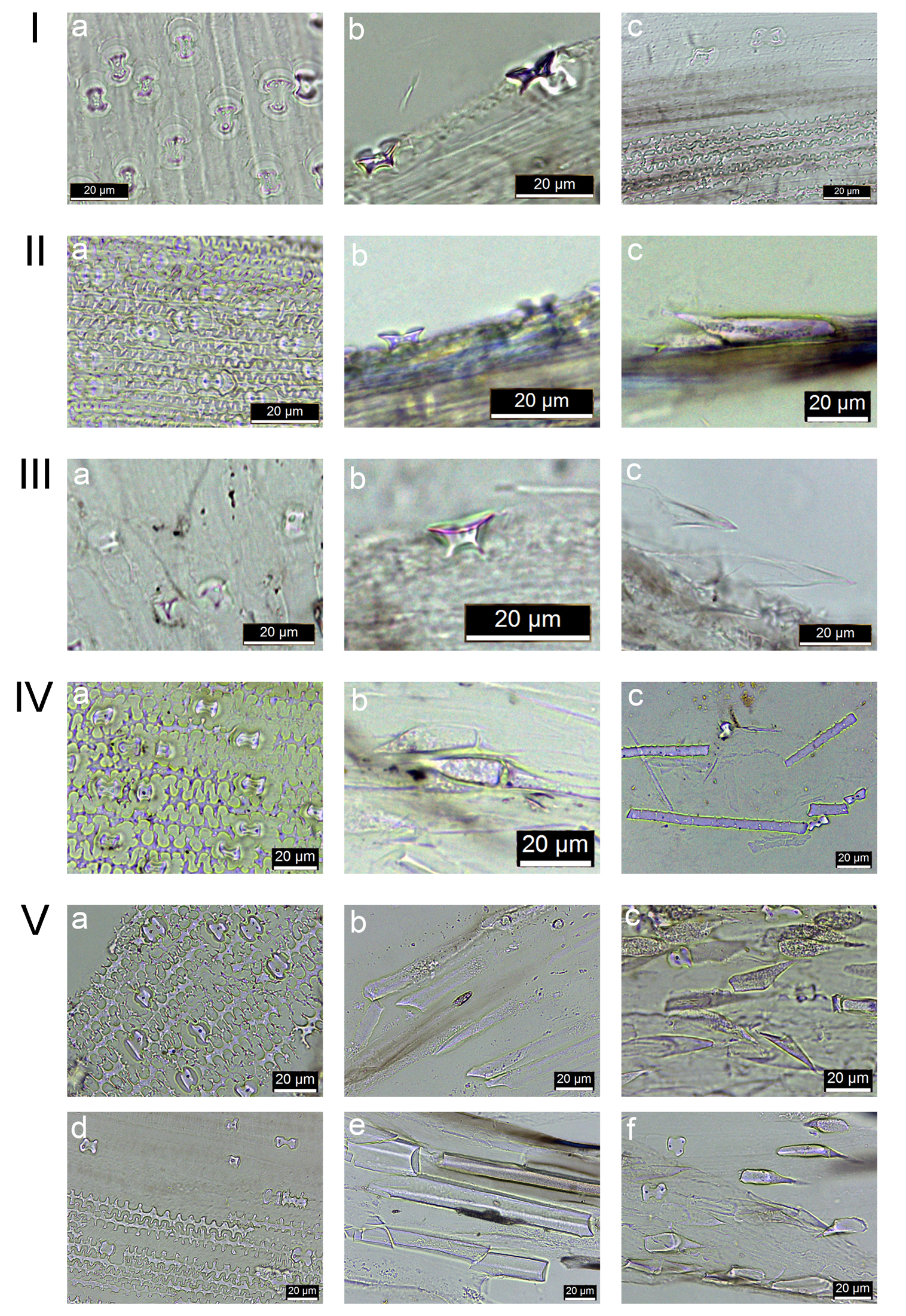

Supplement: Supplementary file 5 [file Image_4.jpeg]

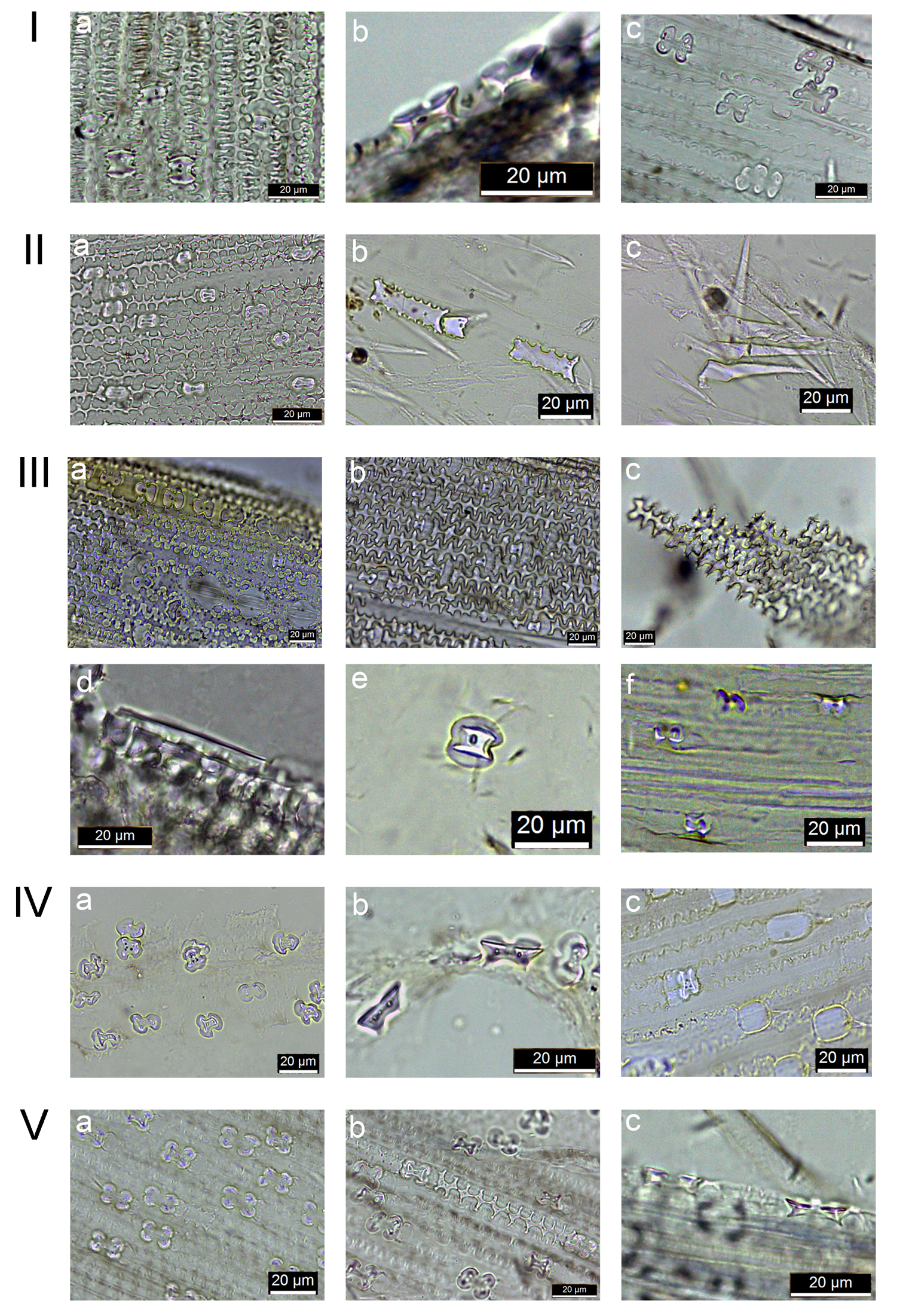

Supplement: Supplementary file 6 [file Image_5.jpeg]

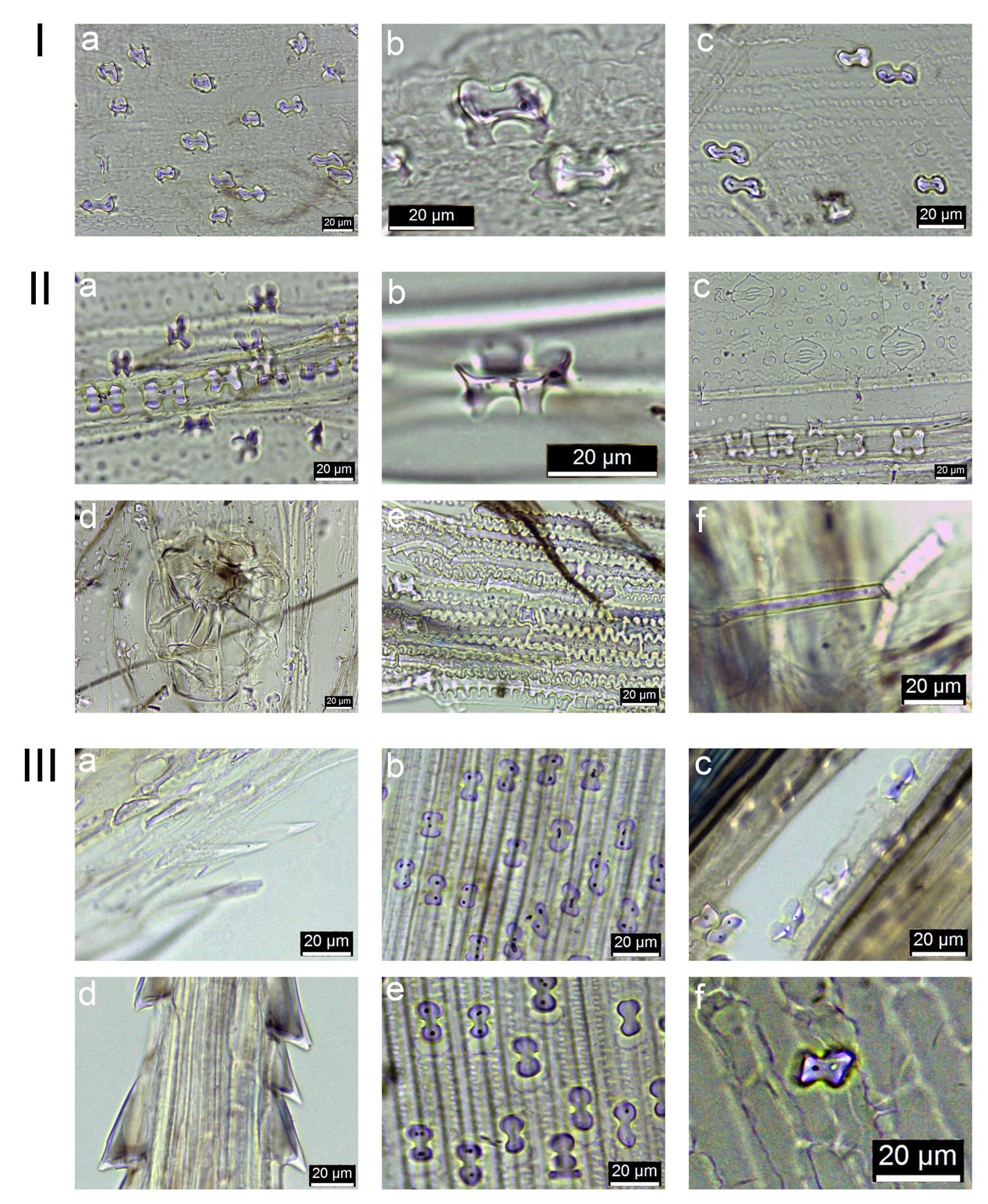

Supplement: Supplementary file 7 [file Image_6.jpeg]

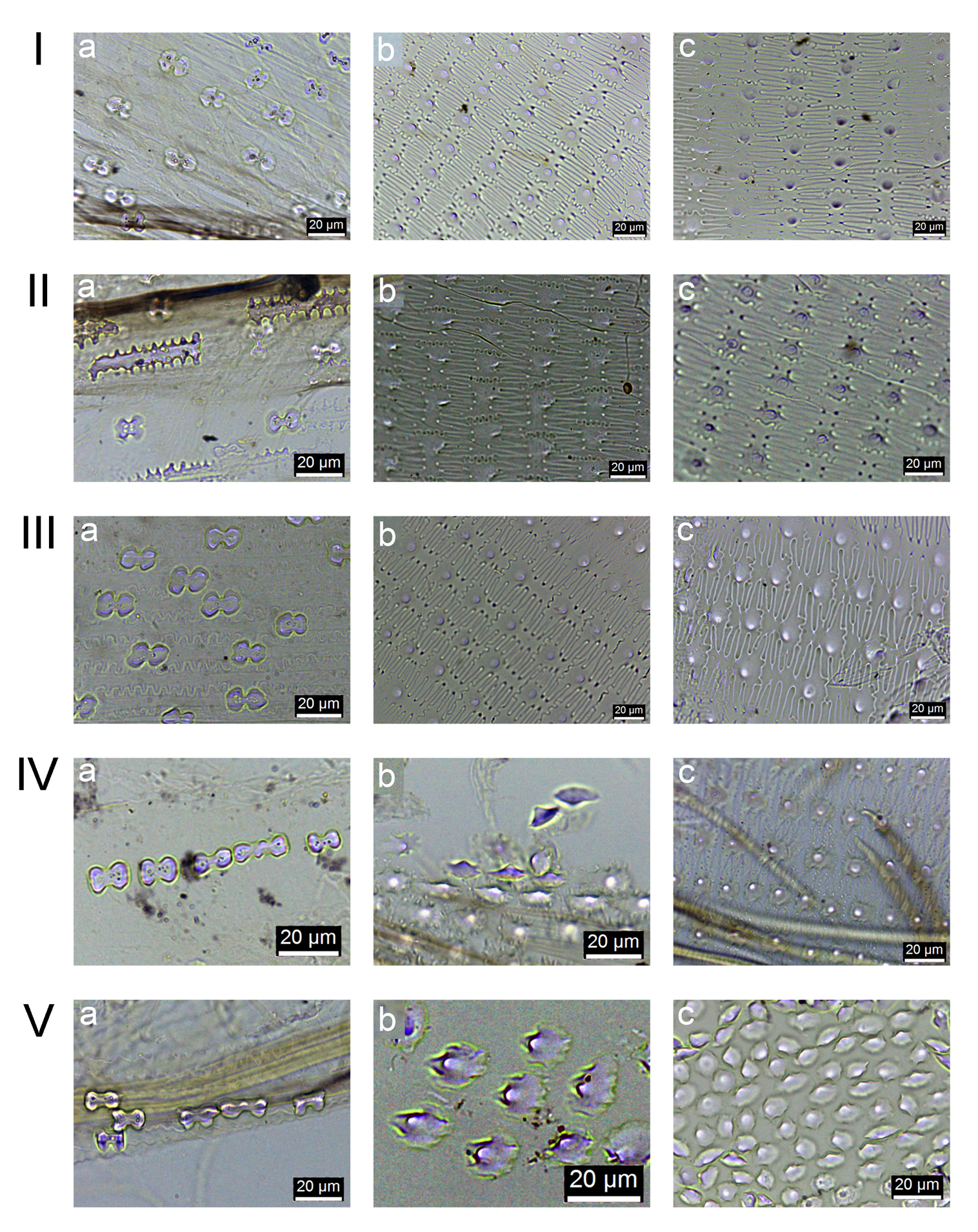

Supplement: Supplementary file 8 [file Image_7.jpeg]

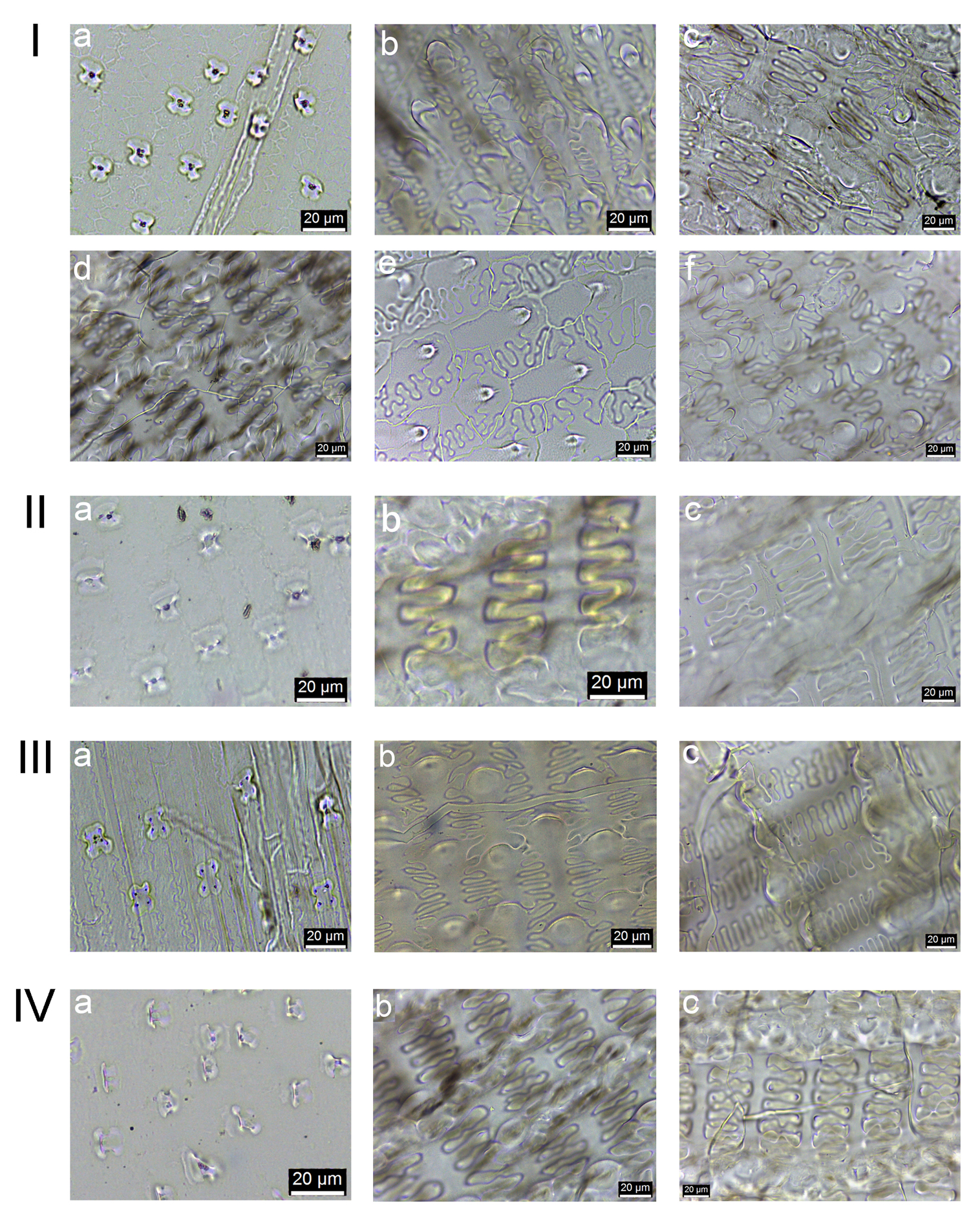

Supplement: Supplementary file 9 [file Image_8.jpeg]

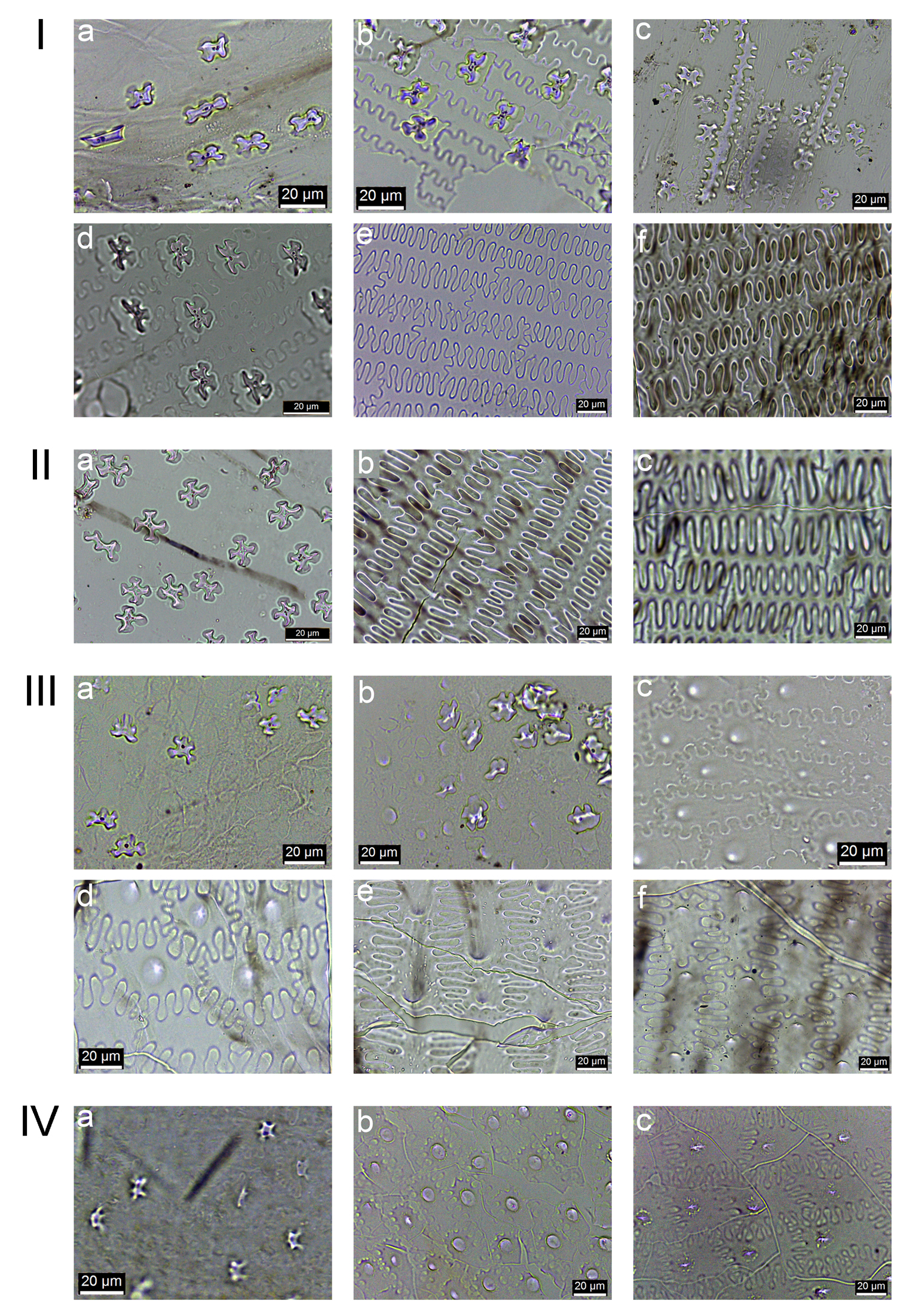

Supplement: Supplementary file 10 [file Image_9.jpeg]
